# Anatomy of Scientific Evolution

**DOI:** 10.1371/journal.pone.0117388

**Published:** 2015-02-11

**Authors:** Jinhyuk Yun, Pan-Jun Kim, Hawoong Jeong

**Affiliations:** 1 Department of Physics, Korea Advanced Institute of Science and Technology, Daejeon, Republic of Korea; 2 Asia Pacific Center for Theoretical Physics, Pohang, Republic of Korea; 3 Department of Physics, Pohang University of Science and Technology, Pohang, Republic of Korea; 4 Institute for the BioCentury, Korea Advanced Institute of Science and Technology, Daejeon, Republic of Korea

## Abstract

The quest for historically impactful science and technology provides invaluable insight into the innovation dynamics of human society, yet many studies are limited to qualitative and small-scale approaches. Here, we investigate scientific evolution through systematic analysis of a massive corpus of digitized English texts between 1800 and 2008. Our analysis reveals great predictability for long-prevailing scientific concepts based on the levels of their prior usage. Interestingly, once a threshold of early adoption rates is passed even slightly, scientific concepts can exhibit sudden leaps in their eventual lifetimes. We developed a mechanistic model to account for such results, indicating that slowly-but-commonly adopted science and technology surprisingly tend to have higher innate strength than fast-and-commonly adopted ones. The model prediction for disciplines other than science was also well verified. Our approach sheds light on unbiased and quantitative analysis of scientific evolution in society, and may provide a useful basis for policy-making.

## Introduction

The history of humankind can be summarized in a series of keywords. From the Palaeolithic Age of stone tools to the Information Age of digital technology, science and technology have played a fundamental role behind keywords such as stone, metal, type printing, internal combustion engine, and Internet. To gain a better understanding of human history, numerous intellectuals have explored innovations in science and technology, e.g., science historians like Thomas Kuhn [1] and futurists like Alvin Toffler [2]. Despite the significant contributions of such endeavours, they are essentially derived from qualitative approaches based on individual’s accumulated knowledge, and thus necessitate complementary methodology with a more quantitative and unbiased focus. In another aspect, some scientists have developed statistical measures of scientific impact based on paper citations. Although these measures can quantify the impact of papers [3], authors [4–5], and journals [6], they are usually focused on gauging the impact within the research community rather than on society in general. Also, there have been built mathematical models to describe the dynamics of scientific paradigms in the whole society [7], but they instead don’t provide much evidence of empirical support. Here, on the basis of empirical data, we attempt systematic and quantitative analysis of scientific evolution in the whole society.

We supposed that an extensive, digitized collection of documents long produced in society might be suitable for such analysis. *Google Books Ngram Corpus* [8–9] covers 8,116,746 books, ~6% of all books ever printed from all fields of publication between 1506 and 2008. Specifically, the dataset provides information regarding the number of times a given 1-gram or *n*-gram occurred in the books over time. Here, a 1-gram is a string of characters uninterrupted by a space, e.g., a word or number. An *n*-gram is a sequence of 1-grams, e.g., a phrase with three words is a 3-gram. For simplicity, we focused only on 1-grams from the corpus of English books. We calculated the relative frequency of each 1-gram defined as the number of instances of the 1-gram in a given year divided by the total number of 1-grams in the corpus in that same year. The frequency, therefore, represents how widely a given 1-gram was adopted in the public. In addition, to obtain sufficient statistical power for the analysis, we restricted our study to the years after 1800, when at least 70 million words were available each year. Because the dataset itself doesn’t provide information regarding which 1-grams are terminologies for science and technology, we identified them with a reference set of scientific and technological words collected from various sources (7,588 words obtained from a science dictionary, scientific journals, and patents; see Materials and Methods). Multiple inflectional forms with a given word stem, such as singular and plural, were integrated systematically when we counted the 1-gram frequency [10]. Because polysemy and synonymy may affect the frequency profiles [11] and thus mislead our analysis, we tried to minimize the presence of the corresponding words amongst our scientific and technological words (Supplementary Methods and Table B, Table C, Table D, Table E, and Table G in S1 File). We further assumed the frequency of a given scientific or technological word to be an estimate of how widely the actual scientific concept was adopted in society (Supplementary Methods in S1 File). All these procedures allowed us to monitor quantitatively the trajectories of science and technology over the years reflected by the frequency profiles.

One clear advantage of investigating such two-centuries-long data, not available from usual online resources with much shorter periods, is that scientific concepts that became widespread after a lag of enormous time could be identified. For example, “biofuel” and “toxicologist” spent 58 and 166 years, respectively, becoming widely used words. Society’s response to a new scientific concept is not always immediate. The origin and significance of such ‘late bloomers’ are discussed later.

## Results

### Characterization and classification of word trajectories

**Fig 1:**
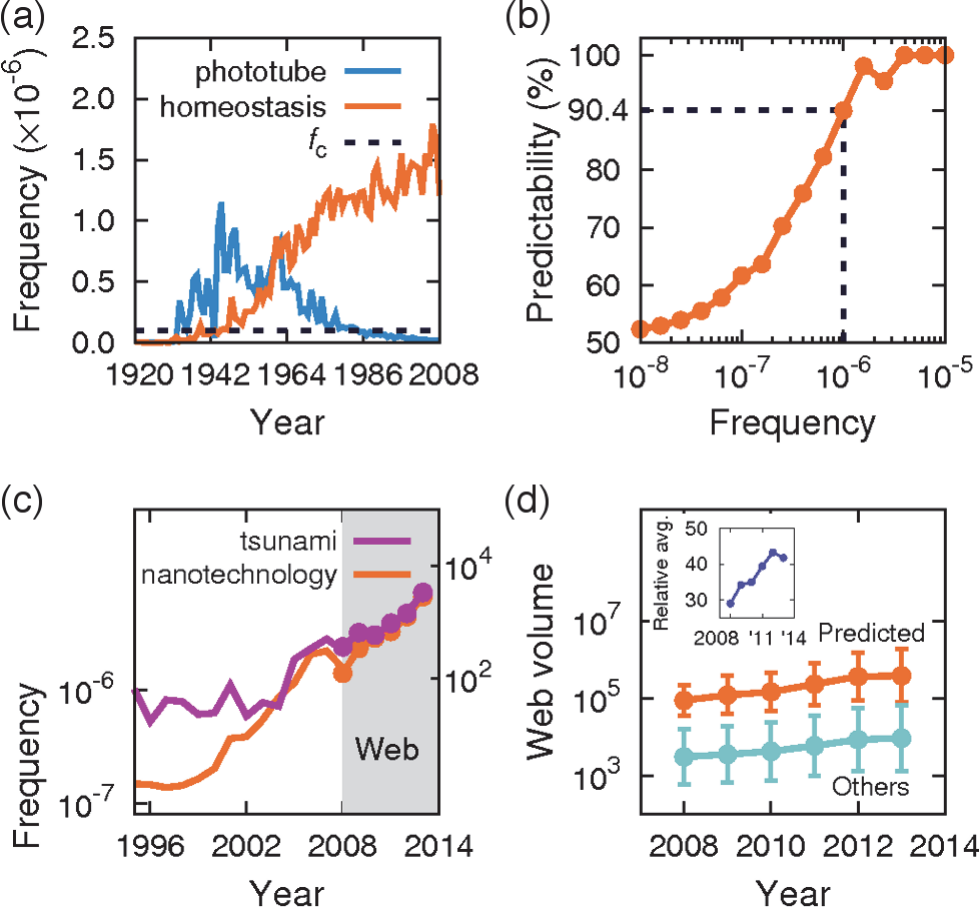
Classification of scientific words and predictability for long-lasting adoption. (a) Examples of type-I and type-II scientific words. The vertical axis represents frequency over the years and *f*_*c*_ is a cutoff frequency used for measuring lifetime. (b) Predictability for type-II (precision of prediction), which is defined as the fraction of type-II among scientific words that passed a particular frequency on the horizontal axis before 1920. (c) Examples of scientific words predicted to be future type-II. From 2008, the shaded area is for the outcomes of the Google web search engine: the right vertical axis represents webpage volumes updated annually, normalized by the geometric mean over random scientific words (Supplementary Methods in S1 File). Matching of each frequency and normalized webpage volume in 2008 is for visual guidance, not intended to infer a one-to-one correspondence between the two scales. (d) Webpage volumes updated annually since 2008, for all scientific words predicted as future type-II and for other randomly-selected scientific words (Supplementary Methods in S1 File). Geometric means are plotted along with error bars from geometric standard deviations. (inset) annual ratio of the geometric mean of the predicted type-II to that of the other random scientific words. In (c) and (d), prediction for type-II was made according to the level of frequency passed between 2000 and 2008.

To characterize the trajectory for each 1-gram, we introduce three measures – first passage time, lifetime, and peak. First passage time (FPT) is defined as years it took the frequency to exceed a certain cutoff *f*_c_ since the onset of the 1-gram, capturing how slowly the 1-gram initially spread into society. Lifetime is defined as years between the first and last time of the frequency over the cutoff *f*_c_, indicating how long the 1-gram was commonly adopted by society (see Materials and Methods). Peak is defined as the highest frequency of the 1-gram over the entire time. For FPT and lifetime, we set *f*_c_ = 10^−7^, which roughly corresponds to a typical frequency of 1-grams found in published dictionaries (Figure B in S1 File) [8]. As a result, most 1-grams could be classified into the following three types: type-I includes 1-grams with finite and well-defined lifetimes within the time frame of our data (like “phototube” in Fig. 1A; for a detailed definition of ‘well-defined lifetimes’, see Materials and Methods). Type-II, in contrast, shows a lifetime to a distinctively long extent beyond the time frame, so the exact lifetime cannot yet be determined (like “homeostasis” in Fig. 1A). One may claim that the classification of type-I and type-II is merely based on the limited period of observation allowed in our current dataset, and thus incorrectly divides the continuum of 1-gram profiles. Although we cannot entirely exclude that possibility, Figure D and Figure E in S1 File do show a more fundamental difference between type-I and type-II: the overall frequency distribution of type-II shifts to higher ranges over time, while that of type-I stays almost steady. This intrinsic difference between types-I and –II seems to have a mechanistic ground, as will be discussed later (Figure O in S1 File). Lastly, type-III, unlike types-I and-II, comprises 1-grams that have not reached any frequency higher than *f*_*c*_, and these words were unlikely to meet in our ordinary life.

### Predictability for long-prevailing scientific concepts

The existence of the above three different types of 1-grams raises an intriguing question: can one predict which science and technology will prove to be type-II (long-term successes) based on levels of prior frequency? By calculating the fraction of type-II among scientific words with each level of frequency exceeded before 1920, we found 90.4% were type-II if a frequency of 10^−6^ was passed (*P* = 2.3×10^−20^; the fraction slightly changes if one considers year ≥ 1920 for the frequency being passed. See Supplementary Methods and Figure F in S1 File). Compared with 61.7% and 52.4% that were type-II for those passing the frequency of 10^−7^ and 10^−8^, respectively (Fig. 1B), 90.4% for 10^−6^ is quite noticeable and gives a simple means to predict type-II with high precision based on this frequency of 10^−6^. In 1897, for example, “nitroglycerin” passed the frequency of 10^−6^, and as currently identified as type-II, has been widely applied to explosives and medicines. As expected, the higher the frequency level crossed by scientific words previously, the more likely they are to be type-II (Fig. 1B). Furthermore, for each level of the frequency crossed, scientific words consistently have a larger probability of being type-II than an entire set of 1-grams (including not only scientific words but also the other 1-grams), e.g., the frequency level of 10^−6^ involves 90.4% and 35.1% type-II for scientific words and the entire set of 1-grams, respectively. Motivated by such findings, we can anticipate which contemporary scientific concepts will be type-II in the future based on their frequency level between 2000 and 2008. First, “tsunami”, a series of huge water waves, rushed to the frequency of 2×10^−6^ in 2006. With a 97.1% chance of being type-II (*P* = 3.0×10^−9^), we predict that “tsunami” will hit our society for a long time (Fig. 1C). Although the fate of the word “tsunami” may be somehow affected by the actual incidence of tsunamis in the future, we notice the tsunamis’ socio-economic implications, not just limited by specific tsunami events. Also, “bioethics” crossed the frequency of 1.5×10^−6^ in 2007 and will continue to receive the spotlight according to our expectation [12]. We observe the rapid rise of “nanotechnology” (Fig. 1C) and practical outcomes of biotechnology, such as “biomarker” and “biosensor”. Although not explicit, aging seems to be an important consensus of several rising words such as “osteoarthritis” (degenerative arthritis) and “nephropathy” (kidney disease) [13–14]. Cancer and neurological diseases, partially relevant to aging as well, will also live with us for a long time, according to our prediction (see Table B, Table C, Table D, and Table E in S1 File for the detailed list).

Note that our prediction is based on the 1-gram dataset available up to 2008. To test how accurate the prediction results can be with a separate up-to-date dataset, we obtained the Internet webpage volumes (as a proxy for word usage) updated annually for scientific words between 2008 and 2013 (e.g., Fig. 1C for “tsunami” and “nanotechnology”; see Materials and Methods). Indeed, overall webpage volumes of scientific words predicted as future type-II consistently exceed those of other random scientific words by an order of magnitude in the years between 2008 and 2013 (Fig. 1D). On average, the ratio of such webpage volumes between the type-II-predicted words and the random counterparts even increases by 44.1% from 29.0 to 41.8 in the same period, indicating the divergence between their growth patterns (Fig. 1D inset). We therefore conclude that our prediction works well beyond the time frame of our 1-gram data.

### Tipping point of scientific evolution

**Fig 2:**
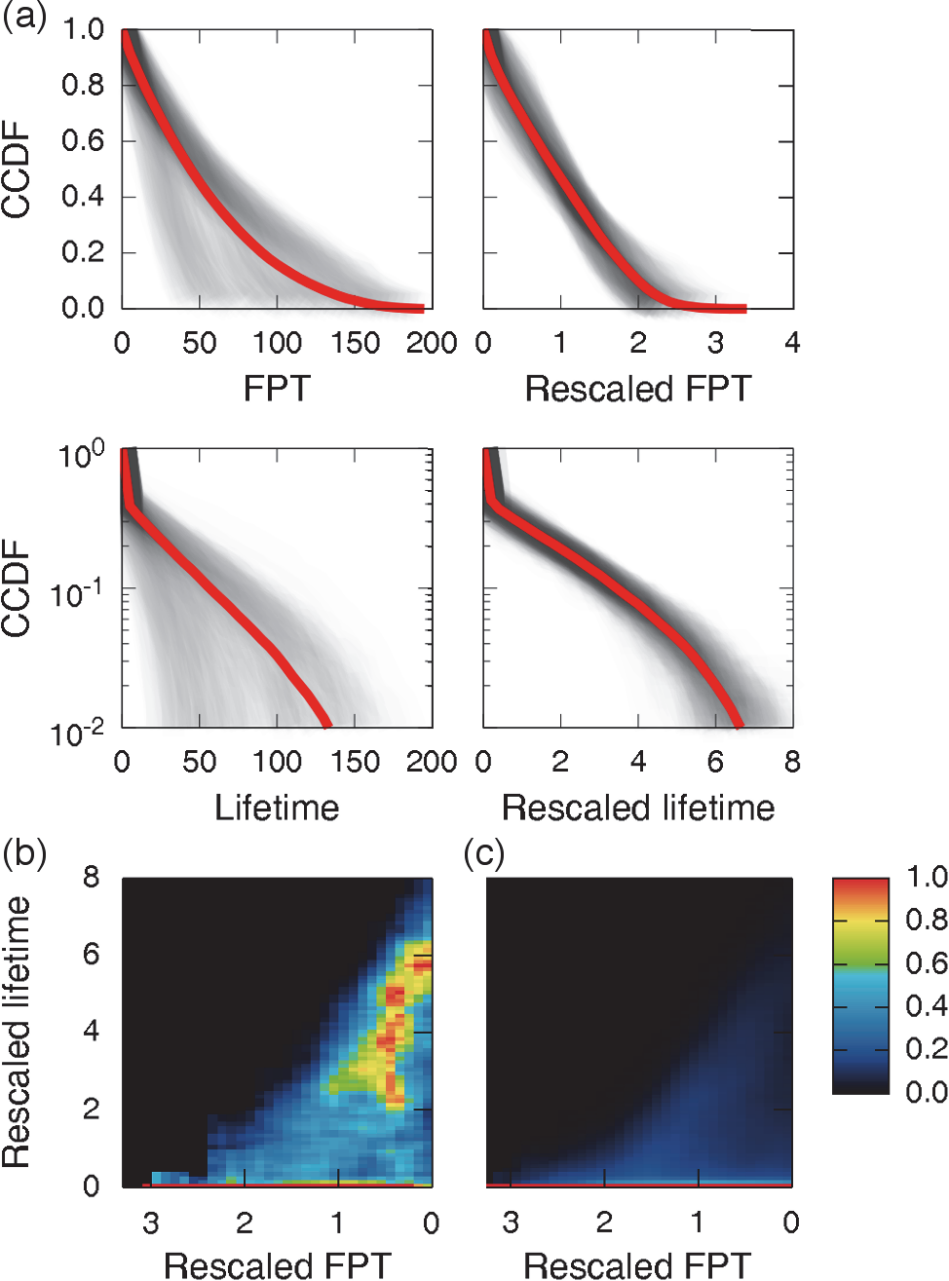
Characteristics of first passage time (FPT) and lifetime. (a) Complementary cumulative distribution functions (CCDFs) of FPT, lifetime, and their rescaled values for type-I 1-grams. Shaded areas correspond to the CCDFs with each for 1-grams from the same year of birth. Each red line denotes the CCDF for all 1-grams aggregated from different years of birth. (b, c) Density plot between rescaled FPT and lifetime in the type-I case, for scientific words (b) or for an entire set of 1-grams (c). We hereafter call the rescaled FPT and lifetime from the data simply FPT and lifetime. Each spot is coloured according to the density of 1-grams at the corresponding FPT and lifetime. Specifically, for each value of FPT, we normalized every density relative to the maximum across lifetime, and according to this adjusted density, coloured the spot following the scale bar on the rightmost side (see Supplementary Methods in S1 File).

In order to proceed to in-depth analysis of scientific evolution, we stress the fact that the overall FPT and lifetime of 1-grams were getting shorter over the past years (Figure G and Figure H in S1 File), indicating the acceleration of cultural turnover over time as reported in the original study of *Google Books Ngram Corpus* [8]. This global effect of accelerating ‘time’ itself makes it unfair to directly compare FPTs or lifetimes many years apart. To compensate for such accelerating effect, we propose the rescaled measures of FPT and lifetime, which now lead to very similar patterns across years (Materials and Methods; see Fig. 2A and Figure G in S1 File). Therefore, the rescaled measures are almost free from the temporal acceleration effect, making it possible to recruit numerous 1-grams from different years into the same place for analysis. For FPT and lifetime from the data, we hereafter use their rescaled values unless specified.

A logical step forward is to search for any possible interplay between FPT and lifetime in scientific evolution, regarding the long-term effect of initial adoption rates inversely captured by FPT. One can suppose that lifetime varies gradually as a function of FPT through the progressive long-term effect of FPT. Unexpectedly, we discover that type-I scientific words undergo a sudden transition from unimodality to bimodality in their lifetime at a particular value of the FPT. The bimodality at this transition (FPT~1.2) is characterized by two prominent lifetimes of ~2.0 and < 0.1, while the unimodality is characterized by < 0.1 (see Fig. 2B). In other words, once initial adoption rates are even slightly higher than a particular value, type-I scientific words may possibly exhibit sudden leaps in their eventual lifetimes (*P* = 4.3×10^−47^). However, an entire set of type-I 1-grams, which includes not only scientific words but also the other 1-grams comprehensively, doesn’t show such behavior (Fig. 2C). Besides the case of FPT, an increase in peak leads to a similar transition of lifetime for scientific words, but does so for an entire set of 1-grams barely at much larger peak, 11.3 times as large as scientific words (Figure J in S1 File). Taken together, the results demonstrate that the temporal evolution of science and technology is subject to an *abrupt* transition at a threshold or ‘tipping point’. The possible mechanism behind the transition will be addressed below, through our mathematical modeling.

### Mechanistic model of scientific evolution

To understand the underlying dynamics of the observed patterns, we start by identifying three key factors that drive the adoption of science and technology. First, there is preferential adoption. People are more likely to adopt already widespread, popular items than to adopt less popular ones because of a variety of psychological, sociological, and economical reasons [15–16], possibly resulting in the rich-get-richer phenomena of innovation spread. Second, the adoption of innovations may also be affected by homophily [17], according to which innovations are more likely to spread among people with similar interests or similar professions. Therefore, newly-introduced science and technology are likely to be shared easily within the scientific community itself rather than between the scientific community and the other communities. Third, every innovative item has its own intrinsic value or fitness, which confers an inherent difference to the item’s adoption rate from that of another [18–19]. Here we bypass the need to dissect fitness into its detailed constituents, and rather view it as a collective quantity accounting for people’s response to an item.

By incorporating the above three factors, we created a mechanistic model of innovation spread. The model comprises *N* agents where the individual agents represent various forms of social units. Agents can invent and adopt items, and the items are transmitted stochastically [20] from agent to agent. Every item is classified into either the scientific category or other, and every agent has the capacity to adopt a total of *L* different items. We further assume that the number of agents, who adopt a particular item, is correlated with that item’s frequency in the 1-gram dataset. In other words, the word frequency in the 1-gram dataset is modeled by the item’s prevalence among the agents. In the model, the items are adopted through a pre-assigned network between agents as follows. One agent *i* accepts an item *q*_*j*_ of its nearest neighbour agent *j* in the network provided that agent *i* has never adopted the item *q*_*j*_ before [7]. The item *q*_*j*_ subsequently replaces the item *q*_*i*_ of the closest category in the agent *i* with the following probability:

$$P(q_{i},q_{j},i,j)=f(\lambda_{q_{j}}-\lambda_{q_{i}})\times p(q_{j},i)\times p(q_{j},j)$$ (1)

where *λ_q_i(j)__* is the item *q_i(j)_*’s fitness, *f* (*λ_q_j__* – *λ_q_i__*) is an increasing function of the fitness difference *λ_q_j__* – *λ_q_i__*, and *p*(*q*_*j*_, *i*)×*p*(*q*_*j*_, *j*) reflects the effect of preferential adoption and homophily. Specifically, *p*(*q*_*j*_, *i*) takes the following functional form:

$$p(q_{j},i)=\sqrt{\frac{\sum_{r}\delta (q_{j},r)w(|\varepsilon_{i}-\varepsilon_{r}|)}{\sum_{r}w(|\varepsilon_{i}-\varepsilon_{r}|)}}$$ (2)

where *δ*(*q*_*j*_, *r*) = 1 if agent *r* has the item *q*_*j*_, otherwise, *δ*(*q*_*j*_, *r*) = 0, and *ε*_*i*(*r*)_ = 1 if agent *i* (*r*) belongs to the scientific community, otherwise, *ε*_*i*(*r*)_ = 0. *w*(|*ε*_*i*_ – *ε*_*r*_|) is a decreasing function of |*ε*_*i*_ – *ε*_*r*_|. The frequency of an item is defined as the ratio of the item’s copy number to the total counts of items (= *N*×*L*) in the system. For more details of the model, see Materials and Methods.

**Fig 3:**
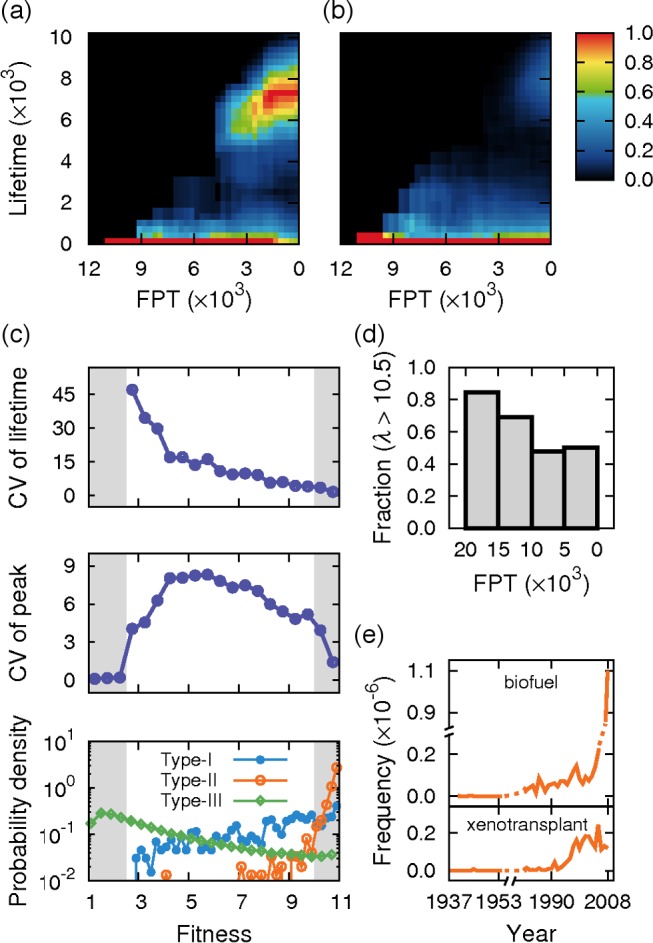
Model simulations and late bloomers. (a, b) Density plot between FPT and lifetime in the type-I case, for scientific items (a) or else (b) from the model simulation. Coloured in the same way as Fig. 2B and Fig. 2C. (c) Uncertainty in the long-term fate of science and technology. For each value of fitness, plotted are the coefficient of variation (CV) of lifetime (top), CV of peak (middle), and the probability densities of types-I, -II, and-III (bottom). CVs of lifetime and peak were obtained from all three types by defining the lifetime of type-III as zero. The shaded area on the top left side includes only type-III, clearly having a uniform lifetime (of zero) in spite of ill-defined CV. Therefore, in the top and middle panels, intermediate fitness shows larger uncertainty of lifetime and peak than low and high fitness of the shaded areas. (d) For each range of FPT, the fraction of high fitness (fitness *λ* >10.5) among scientific items with well-defined finite FPT (i.e., types-I and-II). (e) Empirical examples of late-bloomer scientific words. Both “biofuel” and “xenotransplant” belong to type-II, with ~60 years passed to reach the frequency of 10^−7^ since their birth. The model simulations in (a–d) were performed under the parameters described in Materials and Methods.

For both scientific and other items, the mechanistic model captures essential features of empirical relationship between FPT and lifetime in the type-I case (Fig. 3A and Fig. 3B; compare them with Fig. 2B and Fig. 2C) as well as manifests distinctively long lifetime for type-II (Supplementary Methods and Figure L, Figure M, Figure N, and Figure O in S1 File). Specifically, preferential adoption and homophily are crucial to demonstrate the splits of lifetime into different groups: a separation of type-I and type-II, and an abrupt transition in type-I scientific items. Without preferential adoption and homophily in the model, these splits are hard to observe (Supplementary Methods in S1 File). Fitness is also important in our model. Without fitness, the model fails to produce the diagonal structure that lies in the ranges of rescaled FPT ≤1.2 and rescaled lifetime ≥2.0 in Fig. 2B (Supplementary Methods in S1 File). Therefore, three key components in the model – preferential adoption, homophily, and fitness – are important toward explaining the observed patterns in scientific evolution. Interestingly, according to the model, type-I and type-II scientific items are adopted longer in the opposite places, type-I in the scientific community and type-II in the outer society (Figure O in S1 File).

### Determinism versus contingency, and late bloomers

The accomplishments of our model encourage us to address mechanistic issues in science history otherwise difficult to do. The history of science and technology can be seen from two different viewpoints, determinism versus contingency [21]. Relating to these viewpoints, to what extent does the fitness considered in the model ‘determine’ the success of individual science and technology? Both lifetime and peak, indicators of long-term success of scientific items, increase, on average, as functions of fitness (Figure P in S1 File). However, the average trend itself doesn’t indicate how deterministic it is, and the variability of individual items out of such average trend requires examination. We found that, against the averages at given fitness, lifetime and peak are the most variable at the intermediate level of fitness, while they are less variable, more deterministic at high- and low-level fitness (Fig. 3C). Consistently, we observe that type-II (-III) scientific items have a distribution much biased to high-level (low-level) fitness, making this fitness regime less variable (Fig. 3C).

In addition to lifetime and peak, FPT draws our attention to its relationship with fitness. Because type-III never attains a frequency higher than the cutoff *f*_c_, its FPT is ill-defined and can be regarded as infinite. Type-III, namely having infinite FPT, occupies a larger fraction as fitness gets lower (Fig. 3C). This fact, as well as common intuition, suggests an inverse relation between FPT and fitness for types-I and-II having well-defined finite FPT. Contrary to this expectation, we discover that types-I and-II with long FPT surprisingly tend to have higher fitness than those with short FPT (Fig. 3D and Figure S in S1 File). Indeed, in Fig. 3D, 72.7% of long FPT >10000 are associated with high fitness >10.5, while only 49.6% of shorter FPT are associated with that high fitness (*P* = 5.7×10^−8^). What makes slowly-adopted, long-FPT science and technology have high fitness? The reason, briefly, lies in the fact that high-fitness helps the science resist even long hard times of frequency < *f*_c_, yielding long FPT as well as short FPT. In contrast, low-fitness science is difficult to sustain unless it initially spreads rapidly, either acquiring short FPT or falling to type-III (Figure T in S1 File); ‘late bloomers’ are permitted by high fitness rather than by low fitness. Besides the model results, *Google Books Ngram Corpus* contains a number of actual late bloomers in science and technology. For example, “biofuel” crossed the frequency of 10^−7^ in 2004, 58 years after its birth, involving renewable energy and environmental issues (Fig. 3E) [22]. “isoflavone”, a compound in soybean, required 70 years to reach the same frequency, and is receiving attention for its anti-cancer effects [23]. Also, “toxicologist” had to wait even 166 years until it met a frequency of 10^−7^ in 1975. In medicine, “xenotransplant”, animal tissue or organ transplant in a human patient, was initially believed to work hardly due to immunologic barriers [24], but eventually succeeded in passing a frequency of 10^−7^ in 1997, 61 years after the birth (Fig. 3E). Table G in S1 File presents a more comprehensive list of late bloomers observed in scientific evolution.

### Verification of the model prediction for other disciplines

**Fig 4:**
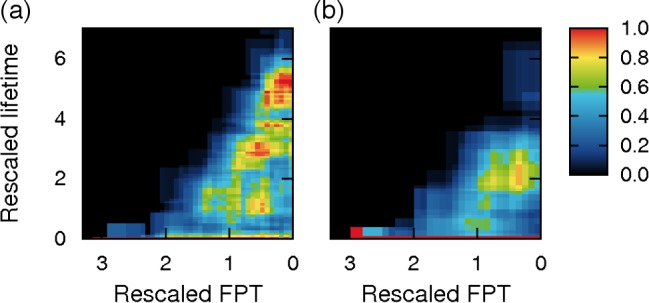
Analysis of other fields: food and art. (a) Data from food and nutrition [25]. (b) Data from art and music [26–27]. In (a) and (b), density plot between rescaled FPT and lifetime for type-I, coloured in the same way as Fig. 2B.

Although our model was primarily intended to account for the observed patterns in scientific evolution, we notice that three key components of the model − preferential adoption, homophily, and fitness − can also be valid for the evolution of other professional fields driven by innovation diffusion between the specialized community and the public. For any fields with these three key components, our model suggests that the relationship between FPT and lifetime for type-I is similar to that shown in Fig. 2B. In this regard, food and art may be good candidate fields to test the prediction. The words in food and art [25–27] indeed follow the predicted patterns in their FPT and lifetime (Fig. 4A and Fig. 4B; *P* = 3.1×10^−9^ for food and 0.018 for art). The results are robust to the exclusion of words overlapping with those analysed for scientific evolution (Figure U in S1 File), supporting the empirical validity of the key components in our model.

## Discussion

In this study, we explored the evolution of science and technology through a massive corpus of digitized English texts over the past two centuries, highlighting the whole society’s influence beyond that of the specialized community (Figure O and Table B, Table B, Table D, Table E, and Table G in S1 File). Scientific evolution is not solely driven by the isolated action of scientists but by the collaboration between scientists and society. We suggest that in-depth analysis into a causal or feedback relation between scientific research and word usage in society may be warranted to enhance the impact of our approach. Also, extending our analysis to *n*-grams with *n*>1 and refining the presented model are left for further study.

Our approach has significant implications for policy-making, especially when complemented by other sophisticated methodologies [28]. Governments and institutions often agonize over the optimal allocation of research resources and incentives to promote good research outcomes [29]. While evaluations for such investments are conventionally based on scholarly outcomes, e.g., the number of publications, patents, and citations, and the reputations from colleagues [3–6], [30], the comprehensive impacts of whole research outcomes outside the professional community have recently begun to be appreciated [31]. Beyond the contents of the printed books that we harnessed in this study, modern information society offers a myriad of online resources to check people’s response to particular science and technology, such as comments in social media, website hits, media exposure, and blog postings [32–34]. In addition, the existence of late bloomers necessitates active consideration of old but recently growing technology for future investment. Going one step forward, if data-driven analysis accompanied by mathematical modelling is judiciously combined with the context-specific perspectives of traditional approaches, the resulting synergy will facilitate an innovative transformation of methodologies in social sciences, humanities, and policy-making.

## Materials and Methods

### Preprocessing of *Google Books Ngram Corpus*

We use the data of *n*-gram counts in the English section of the *Google Books Ngram Corpus* Version 2 [9]. An *n*-gram is a set of *n* successive 1-grams, in which 1-gram is a string of characters uninterrupted by a space. Here, we focus only on 1-grams for simplicity.

The frequency of a 1-gram is defined as the number of occurrences of the 1-gram in a given year divided by the total number of 1-grams in that year. To consider various inflectional forms of words when computing the frequency, we systematically integrated 1-grams by *Porter Stemming Algorithm* [10]. Moreover, we restricted our analysis to the years after 1800 because the quantity of data before 1800 is insufficient to analyse. *Google Books Ngram Corpus* occasionally assigned 1899 or 1905 to books with unknown publication dates [8]. Therefore, for any 1-gram that appeared in the years 1899 and 1905, the frequency was substituted by the average frequency of ±1 years around those years. We also filtered out some 1-grams subjected to possible errors from the optical character recognition (OCR) processes (see Supplementary Methods in S1 File).

### Identification of scientific and technological words

To identify 1-grams belonging to the vocabulary of science and technology, we built a list of science and technology words (as a reference set) from an online science dictionary “*AccessScience”* [35]. However, contemporary dictionary may be rather biased to words that are commonly used today. In order to reduce such bias, we further collected words from various sources covering a wide range of time, including patent grant texts in the *United States Patent and Trademark Office* [36] and titles of articles in scientific journals (Table A in S1 File). We selected only nouns among those words (Supplementary Methods in S1 File). Because frequent usage within the scientific sources was usually for scientific and technological words, we inspected randomly sampled words (≥ 10% coverage for journals, ≥ 1% coverage for patents) along the descending order of usage level within each source, and selected all words of the usage level having at least an 80% chance of being scientific and technological words which are not used in too broad a context. If this cutoff covered all words occurring in that source, then we excluded words that were used only once in the source.

### Characterization of *f*_c_, FPT, lifetime, peak, and different types of 1-grams

We use the cutoff frequency *f*_c_ as the threshold above which a 1-gram can be roughly considered to be common in society. As the quantification of first passage time (FPT) and lifetime depends on *f*_c_, an appropriate choice of *f*_c_ is important, and we choose *f*_c_ = 10^−7^ which roughly corresponds to a typical frequency of 1-grams in published dictionaries [8]. However, our main results do not qualitatively change as long as 10^−8^ ≤ *f*_c_ ≤ 2×10^−7^. For a given 1-gram, first passage time (FPT) is defined as years it took the frequency to cross *f*_c_ since the birth of the 1-gram, lifetime is defined as years between the first and last year of the frequency above *f*_c_, and peak is defined as the highest frequency of the 1-gram over time. Specifically, we define lifetimes only for 1-grams that never exceed the frequency *f*_c_ for at least 10 years until the end time of the data, because they are rarely expected to bounce back (Figure A in S1 File). If the frequency crosses and falls into *f*_*c*_ more than once, we consider the latest event of the falling into *f*_*c*_ as the end of the lifetime.

Most 1-grams can be classified into the following three types. Type-I 1-grams have well-defined finite lifetimes as described above. Type-II shows a lifetime to a distinctively long extent beyond the time frame of the data, so the exact lifetime cannot presently be defined. Finally, type-III includes 1-grams that never had a frequency higher than *f*_c_.

### Internet webpage volume

Because the frequency data from *Google Books Ngram Corpus* is limited until the year 2008, we used the outcomes of the Google web search engine for an alternative up-to-date dataset to test the validity of our type-II prediction results. We collected the Internet webpage volumes updated annually, between the years 2008 and 2013, for the words of our search queries (see Supplementary Methods in S1 File for more details). Because Google itself provides search results based on a stemming algorithm, we searched the singular forms of the words instead of their stems. This work was done manually, regarding the policy of Google, which does not permit automatic search queries by web robots.

### Rescaled measures of FPT and lifetime

We found that the overall FPT and lifetime of 1-grams were getting shorter over the past years (Figure G and Figure H in S1 File). To ‘normalize’ FPT and lifetime from such accelerating effect, we employed their rescaled measures, τ* for FPT and T* for lifetime:

$${\text{τ}}^{*}=\frac{\text{τ}}{{\langle \text{τ}\rangle }_{y}},\;T^{*}=\frac{T}{{\langle T\rangle }_{y}}$$ (3)

where *τ* and *T* are FPT and lifetime of a given 1-gram, respectively, and *τ*_y_ and *T*_y_ are the averages of FPT and lifetime over all 1-grams in type-I with the same year of birth. For FPT and lifetime from the data, we used their rescaled values unless specified.”

### Model construction and simulation

To account for our data analysis results, we built a mechanistic model incorporating preferential adoption, homophily, and fitness, which are described in the main text. The model is based on information spread among *N* agents. Each agent represents an individual or a social cohort, which invents and adopts items. Every agent has the capacity to accommodate a total of *L* different items. The items are transmitted from agent to agent, and we assume that the adopted ranges of such items are projected into the actual usage levels of the corresponding words in our 1-gram dataset [8].

Every agent is assigned *ε*, which characterizes the level of involvement in specialized areas. In general, *ε* can be a vector with real-number components, and here, we only consider the case of scalar binary numbers: *ε* = 1 if the agent belongs to the scientific community, otherwise, *ε* = 0. At the beginning of the simulation, *ε* is assigned to each agent with a chance of *ρ* for *ε* = 1. Once *ε* has been assigned to an agent, either *ε* = 1 or 0, it never changes during the simulation. At every time step, a new item is invented by a randomly-selected agent *m* with probability *α*, and this item belongs to the category following the inventor’s *ε* (i.e., *ε*_*m*_). The item is also assigned fitness *λ*, a positive real number chosen from a given probability distribution [a power-law ~ (*λ*/*λ*_min_)^−*γ*^ for Fig. 3A, Fig. 3B, Fig. 3C, and Fig. 3D; we also considered the Gaussian distribution as described in Supplementary Methods in S1 File]. This new item now replaces one of agent *m*’s old items in the closest category. Next, we randomly select a pair of agents *i* and *j*, among the nearest neighbours in a pre-assigned network structure for innovation spread. Agent *i* accepts agent *j*’s item *q*_*j*_ if agent *i* has never adopted the item *q*_*j*_ before [7], and the item *q*_*j*_ subsequently replaces the item *q*_*i*_ of the closest category in the agent *i* with the probability *P*(*q*_*i*_, *q*_*j*_, *i*, *j*) in equation 1. In the case of Fig. 3A, Fig. 3B, Fig. 3C, and Fig. 3D, the network structure between agents was made according to the Erdős–Rényi model [37], specifically, a *G*(*N,p*_ER_) model, where each agent was randomly connected to another with probability *p*_ER_ [38]. We also considered other network structures with a power-law degree distribution [39], but our main results did not change much against the different network structures. In equation 1 for *P*(*q*_*i*_, *q*_*j*_, *i*, *j*), *f* (*λ_q_j__*–*λ_q_i__*) is an increasing function of the fitness difference *λ_q_j__*–*λ_q_i__*, and *p*(*q*_*j*_, *i*)×*p*(*q*_*j*_, *j*) represents the effect of preferential adoption and homophily. For the case of Fig. 3A, Fig. 3B, Fig. 3C, and Fig. 3D, we employed

$$f(\lambda_{q_{j}}-\lambda_{q_{i}})=\frac{1}{2}+{\left|\frac{\lambda_{q_{j}}-\lambda_{q_{i}}}{10}\right|}^{\beta }\mathrm{sgn}(\lambda_{q_{j}}-\lambda_{q_{i}}).$$ (4)

In equation 2 for *p*(*q*_*j*_, *i*), a square root appears because it makes *p*(*q*_*j*_, *i*)×*p*(*q*_*j*_, *j*) linearly proportional to the population having the item *q*_*j*_ in the case that *ε*’s are identical for all agents. *w*(|*ε*_*i*_ – *ε*_*j*_|) in equation 2 represents the effect of homophily, and is a decreasing function of |*ε*_*i*_ – *ε*_*r*_|. Here, we employed *w*(|*ε*_*i*_ – *ε*_*j*_|) = exp[– (*ε*_*i –*_ *ε*_*j*_)^2^].

At every *N×L* steps of simulation, the frequencies of all items in the system were recorded. The frequency of an item is defined as the ratio of the item’s copy number to the total counts of items (= *N*×*L*) in the system. Here, we use such *N*×*L* steps as the arbitrary unit of time to measure the FPT and lifetime of items. In Fig. 3A, Fig. 3B, Fig. 3C, and Fig. 3D, we present the simulation results with parameters *γ* = 2.0, *β* = 1/4, *N* = 4096, *L* = 10, *p*_ER_ = 0.1024, *ρ* = 0.2, *α* = 0.0001, and *f*_c_ = 0.00025. We identified a range of parameters in which our main results remained robust. See Supplementary Methods in S1 File for full details of our model and parameters.

### Statistical significance test

To test the statistical significance of our results in Fig. 1B, we performed a two-sided *Z*-test under the null hypothesis that there is no association between the frequency level and the probability of type-II. For Fig. 3D, we conducted a similar analysis under the null hypothesis that there is no association between FPT and the fraction of scientific items with fitness > 10.5.

For Fig. 2B, we tested the statistical significance of a sudden leap into ~2.0 in lifetime at FPT ~ 1.2. We constructed a 2×2 contingency table displaying the numbers of the words at FPT ≥ 1.2 and < 1.2, and lifetime ≥ 2.0 and < 2.0. Then, we computed *P*-values based on the Pearson’s Chi-squared test. We also conducted similar analyses for Fig. 3A and Fig. 4.

## Supporting Information

S1 File This file contains Figures 1–U, Tables A–G, and Supplementary Methods. (PDF)
