# Supplementary material for: Anatomy of Scientific Evolution
Source: PLoS One. 2015 Feb 11;10(2):e0117388. doi: 10.1371/journal.pone.0117388 (PMC4325003; doi:10.1371/journal.pone.0117388)
Supplement: S1 File — (PDF) [file pone.0117388.s001.pdf]

Anatomy of scientific evolution

Jinhyuk Yun, Pan-Jun Kim, Hawoong Jeong

Supporting Information

Table of Contents

Supplementary Figures 1

Supplementary Tables.. 22

Supplementary Methods 37

1. Dataset..37

2. Data analysis..38

3. Model description..41

4. Model results 44

5. Late bloomers: effect of fitness on FPT 46

6. Evolution of other fields 47

Supplementary References 48

Supplementary Figures

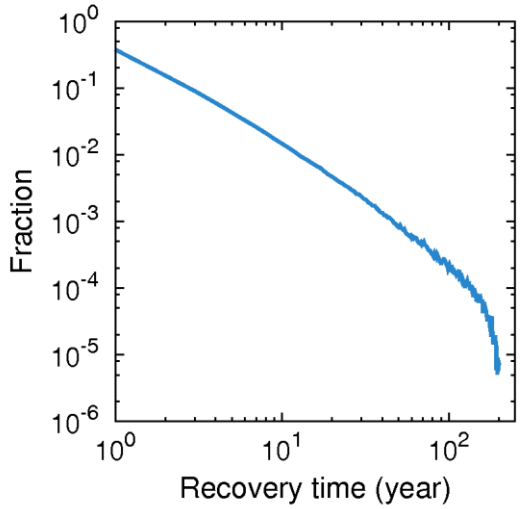

Figure A. Probability distribution of recovery time for 1-gram frequency that fell below 10^{-7} and recovered later. The fraction of recovery time monotonically decreases as the recovery time increases. As a result, most cases (82%) have a recovery time < 10 years.

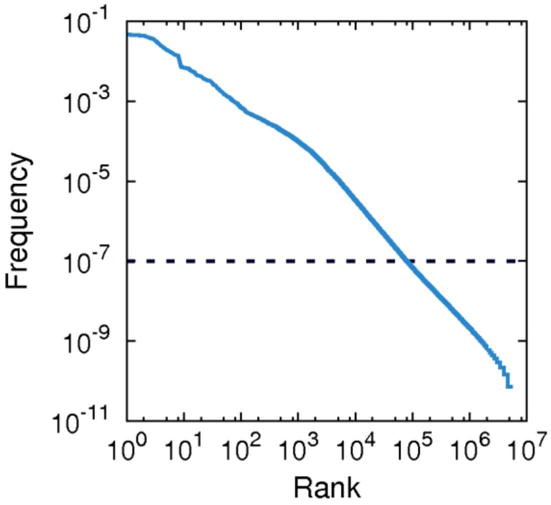

Figure B. Rank-frequency plot for 1-gram stems in the year 2000. Above the frequency of 10^{-7} (dashed line), there are 79,691 stems, which are of the same order as the number of stems in an average published dictionary.

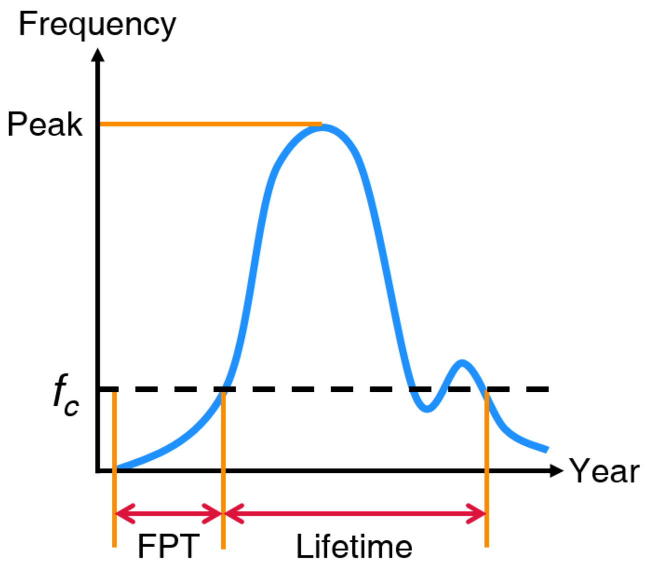

Figure C. Definitions of first passage time (FPT), lifetime, and peak.

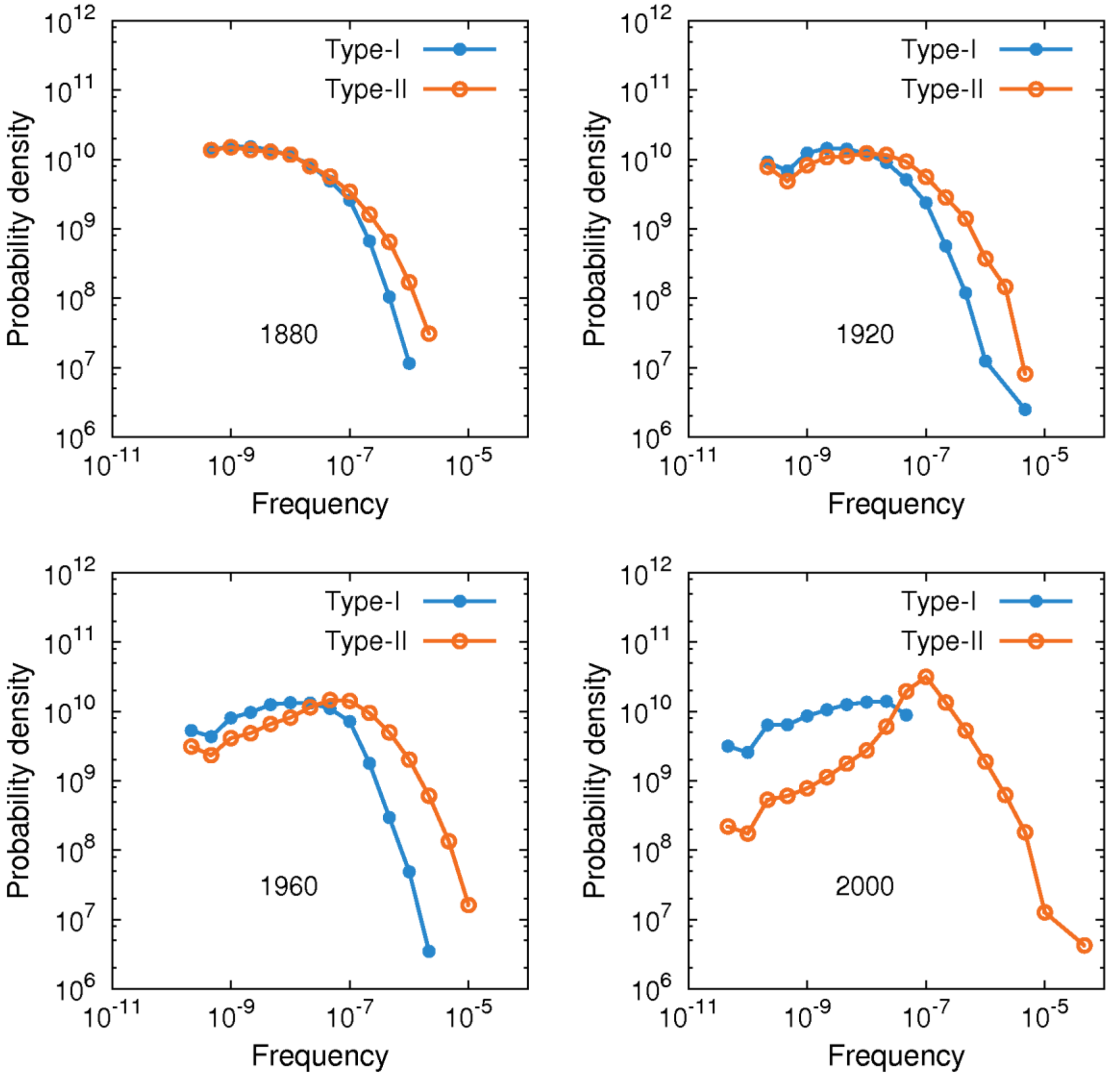

Figure D. Frequency distribution for types-I and -II in each year. In 1880, the probability density functions (PDFs) of types-I and -II almost overlap. As time elapses, the PDF for type-II shifts to higher frequency ranges, while that for type-I stays in almost the same frequency range.

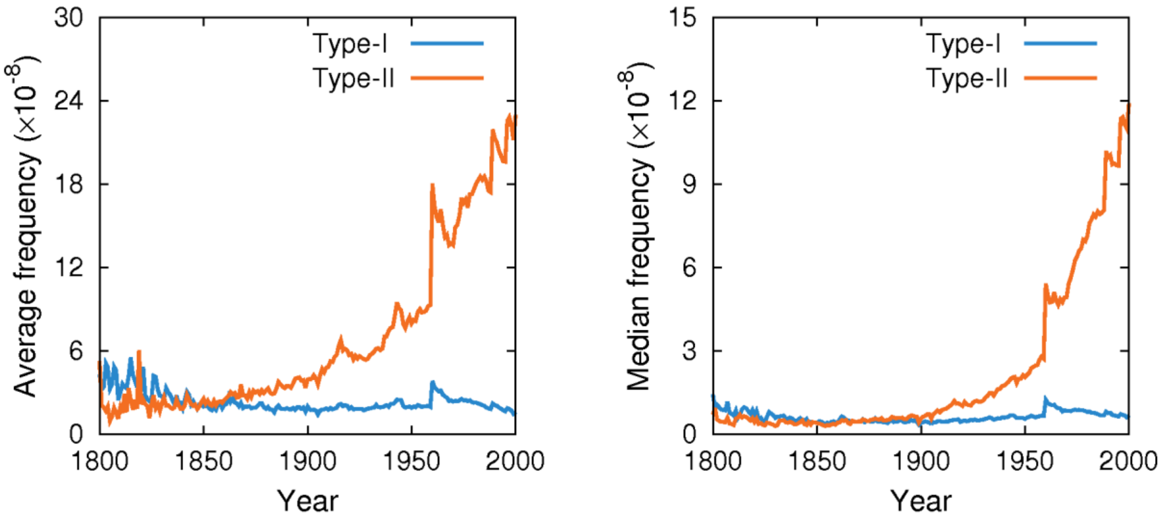

Figure E. Average and median frequencies of 1-grams in types-I and -II over the years. Both the average and median frequencies for type-II increase over time, whereas those for type-I barely change.

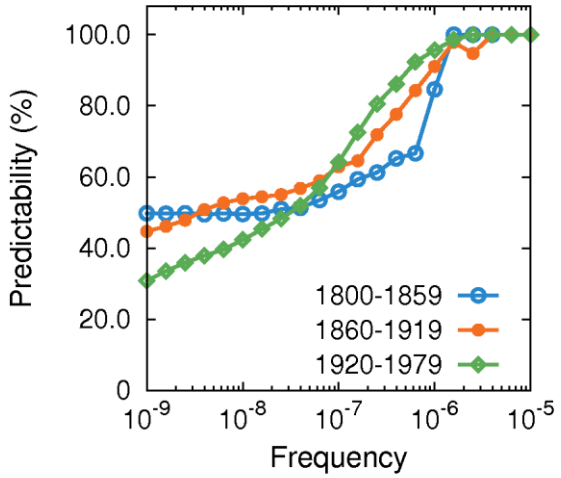

Figure F. Probability that a scientific word turns out to be type-II as of 2008 if it first passed a particular level of frequency on the horizontal axis in a given range of the past years. The higher frequency a scientific word exceeds, the more likely it is of type-II.

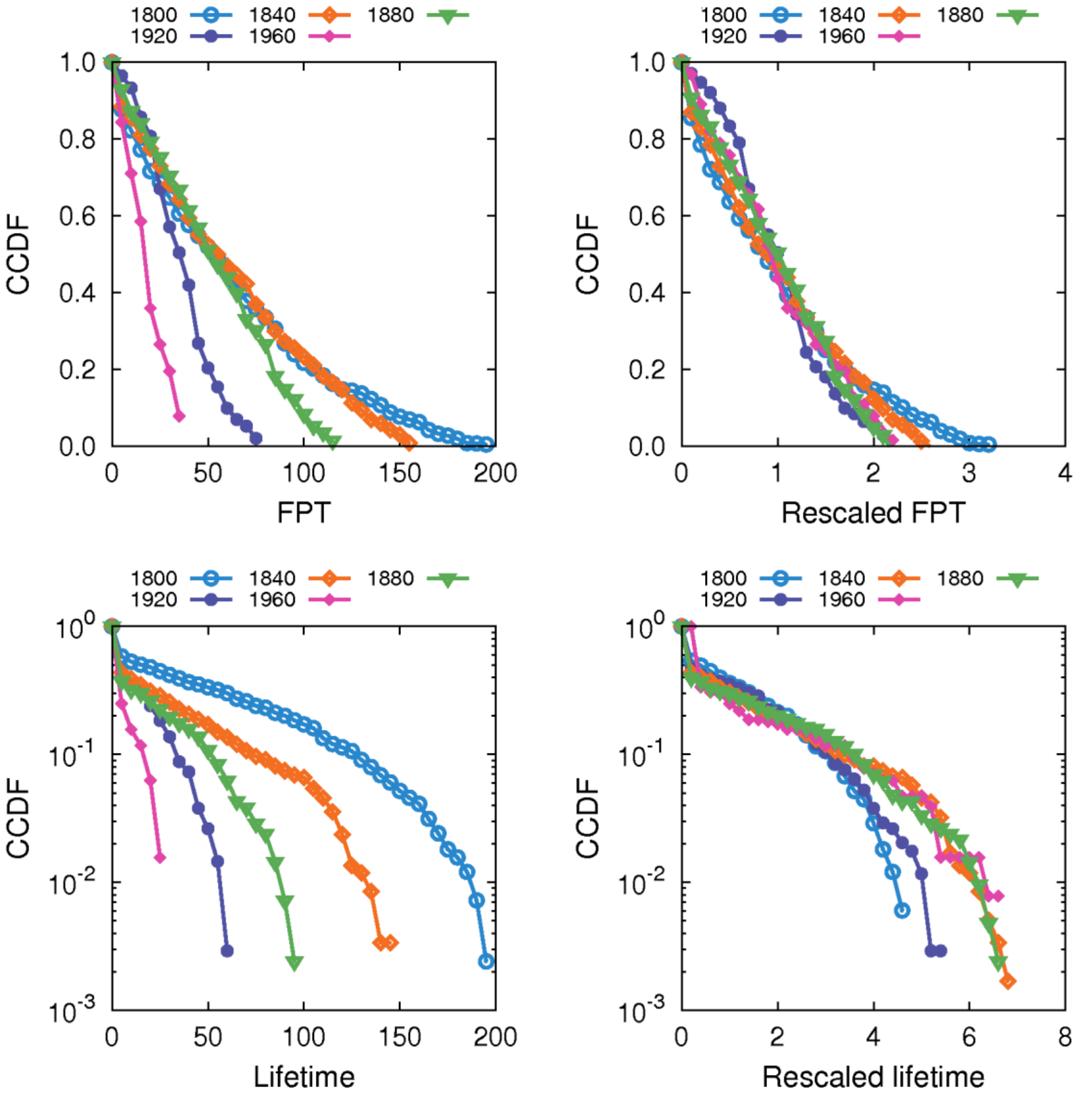

Figure G. Complementary cumulative distribution functions (CCDFs) of FPT, lifetime, and their rescaled values for each set of all type-I 1-grams from the same year of birth.

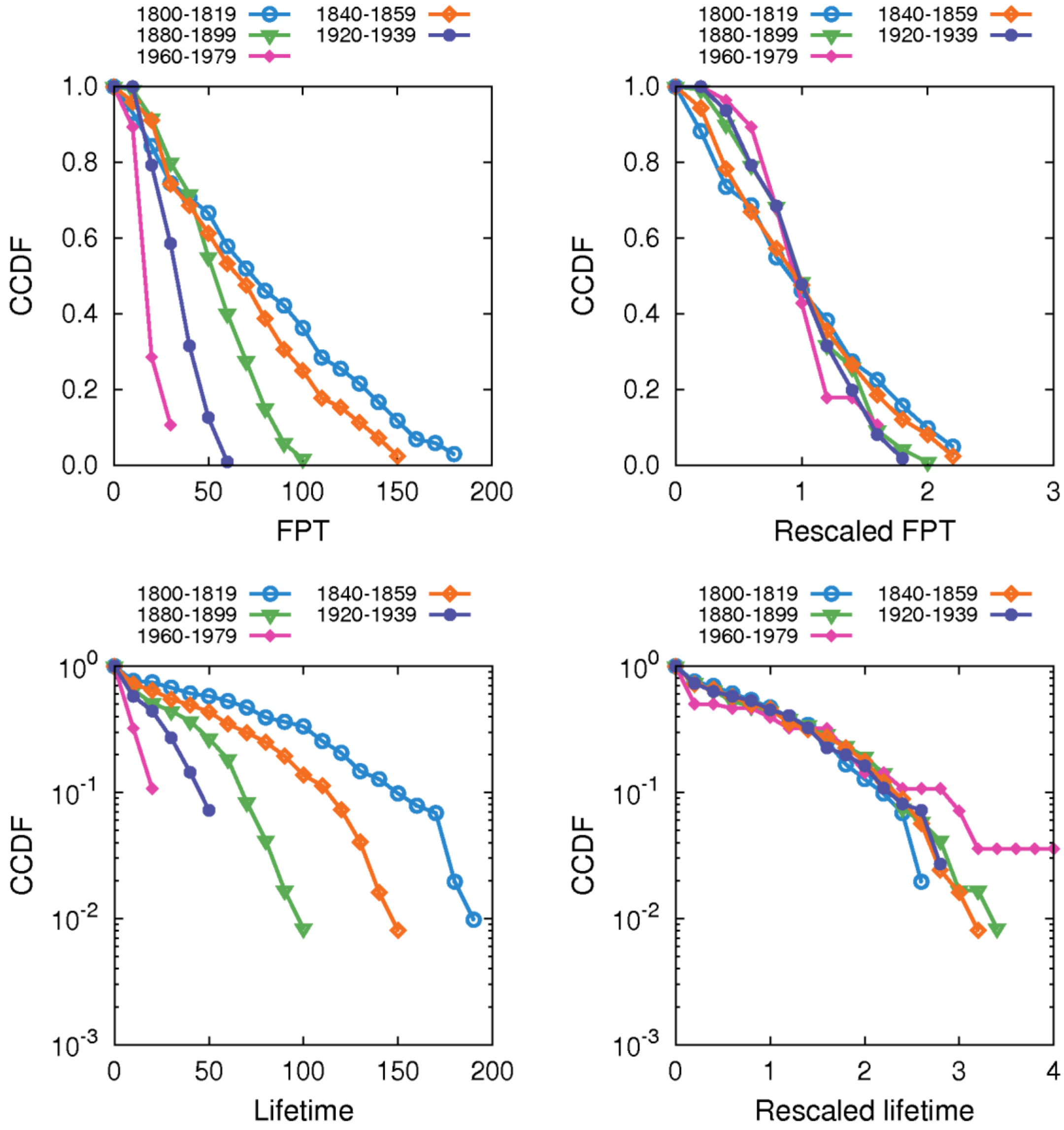

Figure H. Complementary cumulative distribution functions (CCDFs) of FPT, lifetime, and their rescaled values for each set of type-I scientific words from the same year of birth.

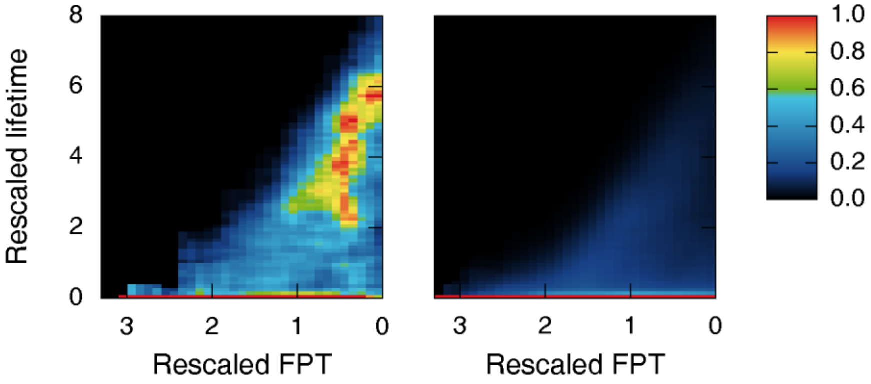

Figure I. Density plot between FPT and lifetime of type-I scientific words (left) and all type-I 1-grams (right). Coloured according to adjusted density, following the scale bar on the rightmost side. $b_x = 0.4$, $b_y = 0.8$, $k_x = 4$, and $k_y = 4$ (see Supplementary Methods).

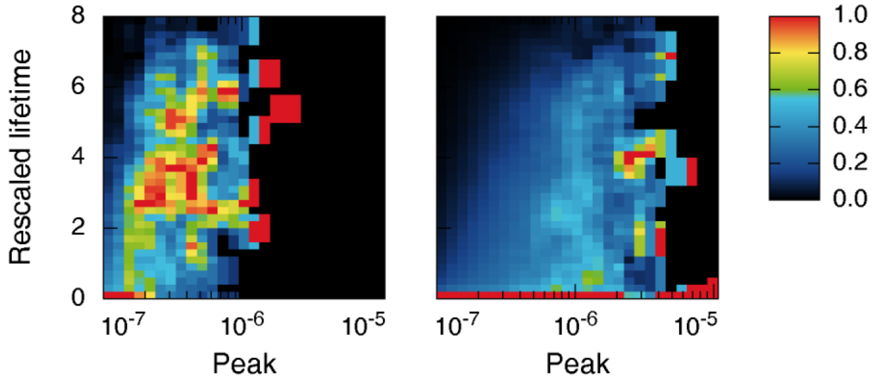

Figure J. Density plot between peak and lifetime of type-I scientific words (left) and all type-I 1-grams (right). Coloured according to adjusted density, following the scale bar on the rightmost side. $b_x = 10^{-7}$, $b_y = 0.4$, $k_x = 4$, $k_y = 4$, and $i_x = 2$ (see Supplementary Methods).

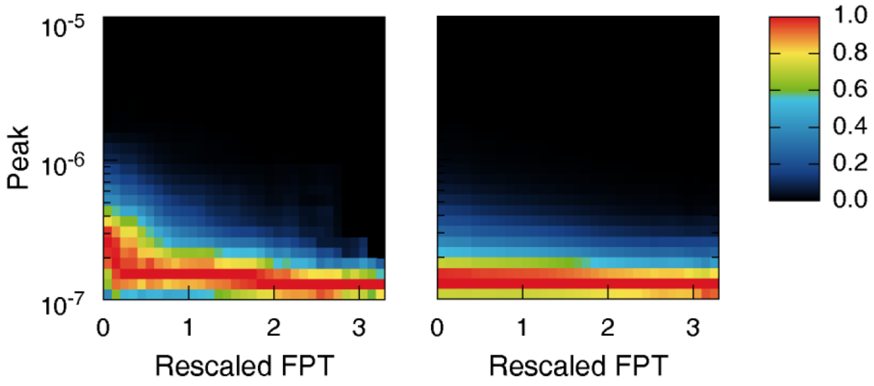

Figure K. Density plot between FPT and peak of type-I scientific words (left) and all type-I 1-grams (right). Coloured according to adjusted density, following the scale bar on the rightmost side. $b_x = 0.8$, $b_y = 10^{-7}$, $k_x = 4$, $k_y = 4$, and $i_y = 2$ (see Supplementary Methods).

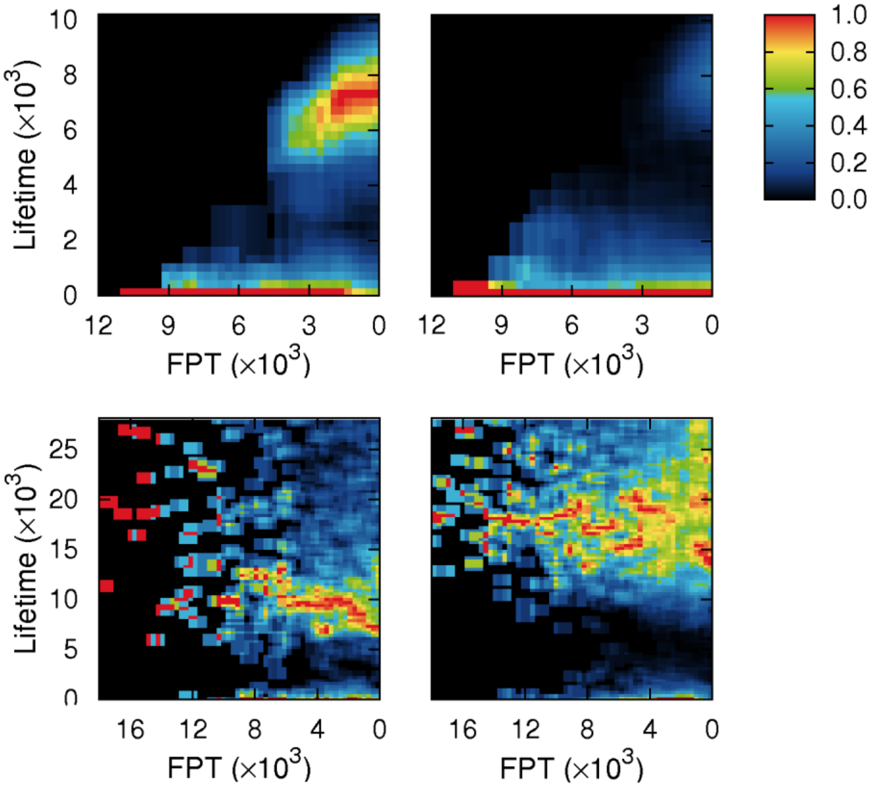

Figure L. Density plot between FPT and lifetime from the simulation with the power-law fitness distribution (γ = 2.0, β = 1/4, N = 4,096, L = 10, p_{ER} = 0.1024, ρ = 0.2, α = 0.0001, f_c = 0.00025). Coloured according to adjusted density, following the scale bar on the rightmost side (b_x = 2,400, b_y = 2,400, k_x = 12, k_y = 12 for the top panel and b_x = 1,600, b_y = 1,600, k_x = 8, k_y = 8 for the bottom panel; see Supplementary Methods). The left panels are for scientific items and the right panels are for the rest. Each top panel is a magnification of the region for FPT + lifetime < 12,000 in the bottom panel, representing the type-I case.

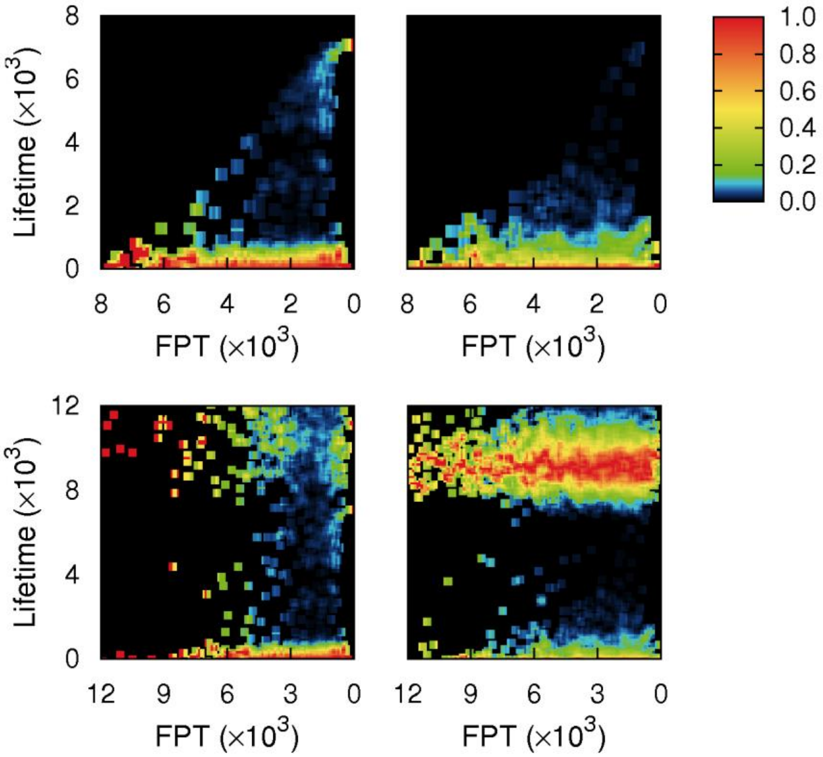

Figure M. Density plot between FPT and lifetime from the simulation with the Gaussian fitness distribution ($\sigma = 0.1$, $N = 4{,}096$, $L = 10$, $p_{\mathrm{ER}} = 0.0064$, $\rho = 0.15$, $\alpha = 0.0001$, $f_{\mathrm{c}} = 0.00025$). Coloured according to adjusted density, following the scale bar on the rightmost side ($b_x = 400$, $b_y = 400$, $k_x = 4$, $k_y = 4$; see Supplementary Methods). The left panels are for scientific items and the right panels are for the rest. Each top panel is a magnification of the region for FPT + lifetime < 8,400 in the bottom panel, representing the type-I case.

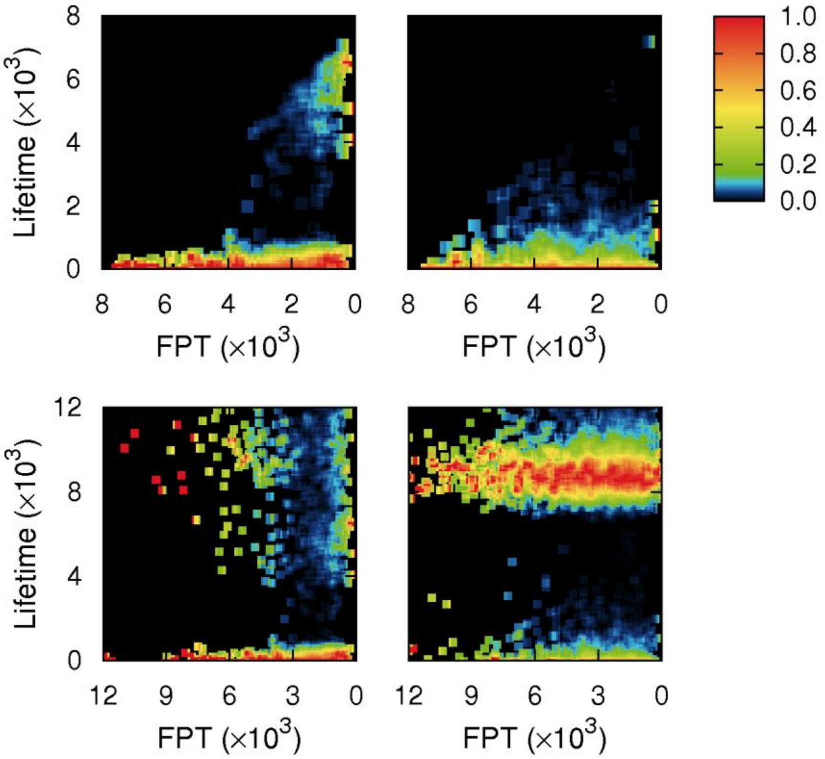

Figure N. Density plot between FPT and lifetime from the simulation with the Dirac delta distribution of fitness (N = 4,096, L = 10, p_{ER} = 0.0064, ρ = 0.15, α = 0.0001, f_{c} = 0.00025). Coloured according to adjusted density, following the scale bar on the rightmost side (b_x = 400, b_y = 400, k_x = 4, k_y = 4; see Supplementary Methods). The left panels are for scientific items and the right panels are for the rest. Each top panel is a magnification of the region for FPT + lifetime < 8,000 in the bottom panel, representing the type-I case.

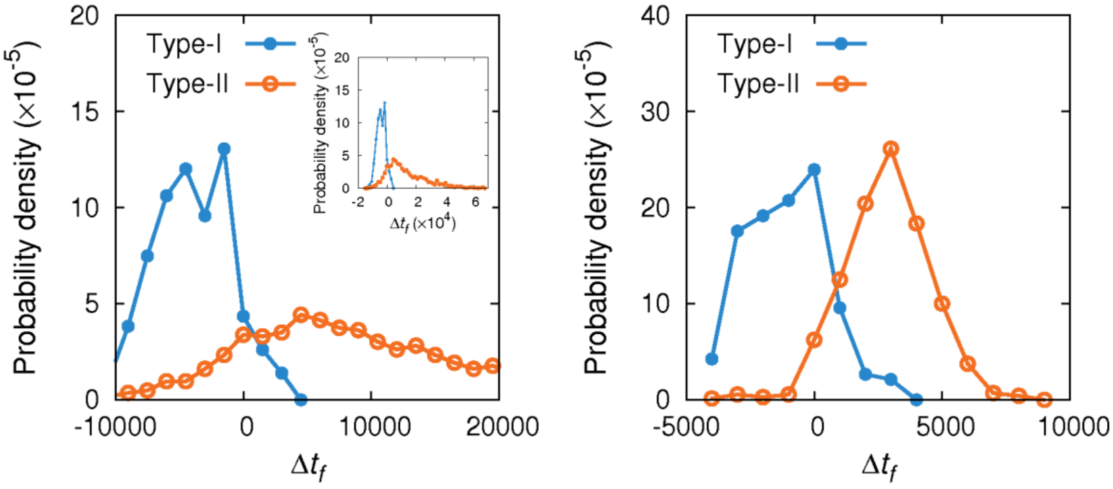

Figure O. Probability distributions of $\Delta t_f = t_f{'} - t_f$ for type-I and type-II scientific items, where $t_f{'}$ (t_f) of each item is the last time that the frequency of the item outside (inside) the scientific community fell below f_c. The left panel is for the power-law fitness distribution (inset for the full range of Δt_f; $\gamma = 2.0$, $\beta = 1/4$, $N = 4{,}096$, $L = 10$, $p_{\underline{ER}} = 0.1024$, $\rho = 0.2$, $\alpha = 0.0001$, $f_c = 0.00025$) and the right panel is for the Gaussian fitness distribution ($\sigma = 0.1$, $N = 4{,}096$, $L = 10$, $p_{ER} = 0.0064$, $\rho = 0.15$, $\alpha = 0.0001$, $f_c = 0.00025$).

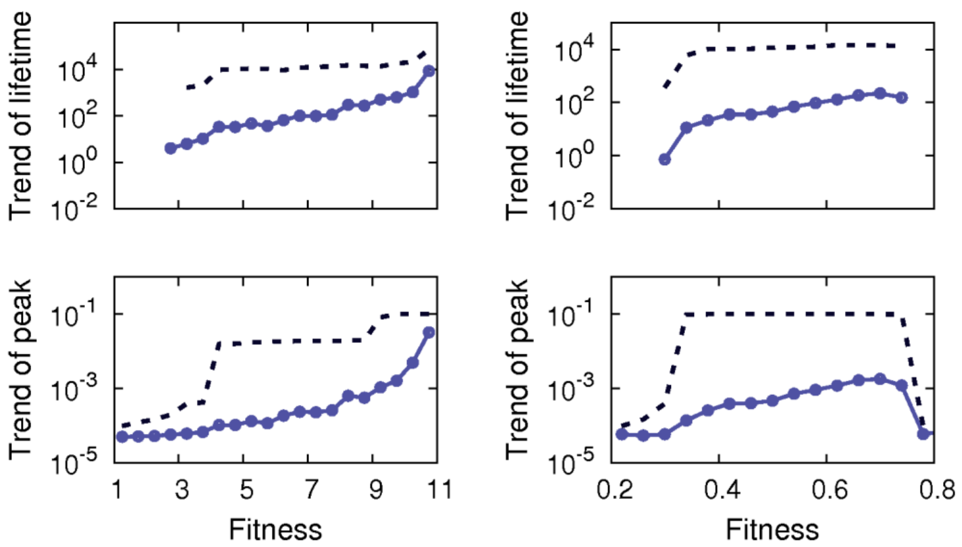

Figure P. Dependency of lifetime and peak of scientific items (for all three types) on fitness. The left panels are for the power-law fitness distribution (γ = 2.0, β = 1/4, N = 4,096, L = 10, p_{ER} = 0.1024, ρ = 0.2, α = 0.0001, f_c = 0.00025) and the right panels are for the Gaussian fitness distribution (σ = 0.1, N = 4,096, L = 10, p_{ER} = 0.0064, ρ = 0.15, α = 0.0001, f_c = 0.00025). Lifetime of type-III was treated as zero for this analysis. For the lifetime and peak at each level of fitness, filled circles represent the averages and dashed lines represent the upper 0.1% of lifetime and peak.

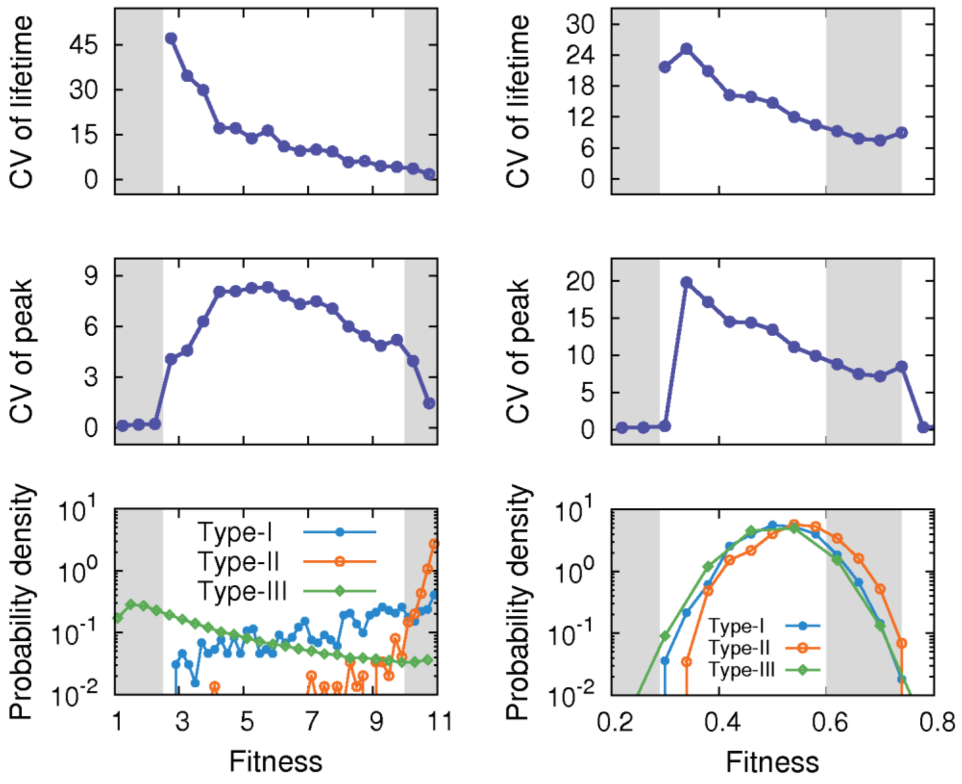

Figure Q. Variability of lifetime and peak of scientific items (for all three types) across fitness. The left panels are for the power-law fitness distribution (γ = 2.0, β = 1/4, N = 4,096, L = 10, p_{ER} = 0.1024, ρ = 0.2, α = 0.0001, f_{c} = 0.00025) and the right panels are for the Gaussian fitness distribution (σ = 0.1, N = 4,096, L = 10, p_{ER} = 0.0064, ρ = 0.15, α = 0.0001, f_{c} = 0.00025). Lifetime of type-III was treated as zero for this analysis. The upper two panels show the coefficients of variation (CVs) of lifetime and peak for each level of fitness, and the bottom panel shows the probability distributions of fitness for different types. The shaded areas indicate the ranges of fitness toward which the distributions of type-II and type-III are biased (type-II for high fitness and type-III for low fitness). In the left shaded areas, CVs of lifetime are ill-defined because all items in the areas belong to type-III with zero lifetime; the variability of lifetime in these areas can be viewed as effectively zero.

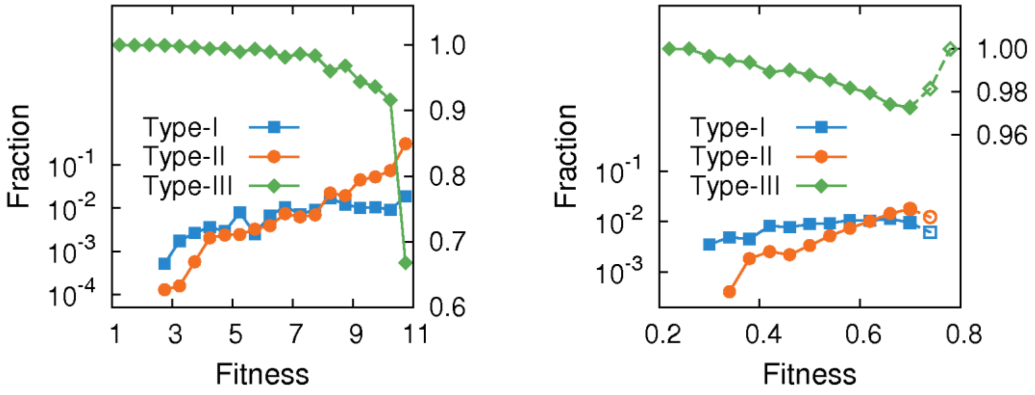

Figure R. Ratios of different types to the total at each level of fitness for scientific items. The left panel is for the power-law fitness distribution (γ = 2.0, β = 1/4, N = 4,096, L = 10, p_{ER} = 0.1024, ρ = 0.2, α = 0.0001, f_c = 0.00025) and the right panel is for the Gaussian fitness distribution (σ = 0.1, N = 4,096, L = 10, p_{ER} = 0.0064, ρ = 0.15, α = 0.0001, f_c = 0.00025). For types-I and -II, plotted in logarithmic scale (left axis). For type-III, plotted in linear scale (right axis). The fractions of type-I and type-II tend to increase as fitness increases, but the slope is steeper for type-II. The fraction of type-III is very high in all ranges of fitness, but slightly increases as fitness decreases (in the right panel, open circles with dashed lines do not have a statistically-meaningful number of items).

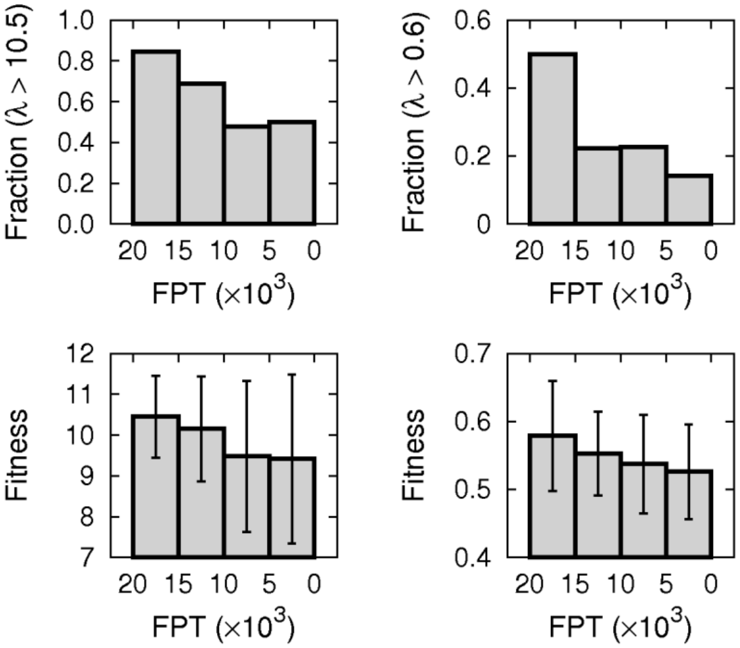

Figure S. Relation between FPT and fitness of types-I and -II scientific items. We checked (left) the case for the power-law distribution of fitness ($\gamma = 2.0$, $\beta = 1/4$, $N = 4{,}096$, $L = 10$, $p_{ER} = 0.1024$, $\rho = 0.2$, $\alpha = 0.0001$, $f_c = 0.00025$) and (right) that for the Gaussian distribution of fitness ($\sigma = 0.1$, $N = 4{,}096$, $L = 10$, $p_{ER} = 0.0064$, $\rho = 0.15$, $\alpha = 0.0001$, $f_c = 0.00025$). The top panels show the fractions of fitness > 10.5 (left) and > 0.6 (right) for each range of FPT. The bottom panels show the averages and the standard deviations of fitness for each range of FPT.

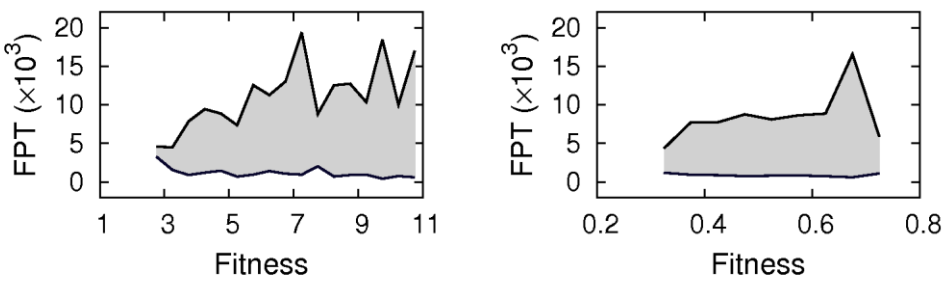

Figure T. Extent of FPT for types-I and -II scientific items. We checked (left) the case for the power-law distribution of fitness (γ = 2.0, β = 1/4, N = 4,096, L = 10, p_{ER} = 0.1024, ρ = 0.2, α = 0.0001, f_c = 0.00025) and (right) that for the Gaussian distribution of fitness (σ = 0.1, N = 4,096, L = 10, p_{ER} = 0.0064, ρ = 0.15, α = 0.0001, f_c = 0.00025). Shaded area is between the upper and lower 1%s in FPT for each fitness.

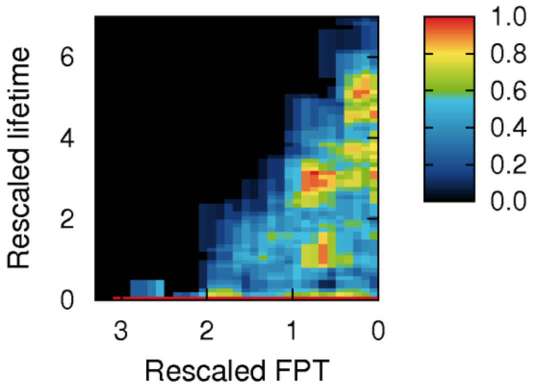

Figure U. Density plot between FPT and lifetime of type-I food and nutritional words without those analysed for scientific evolution. $b_x = 0.6$, $b_y = 1.2$, $k_x = 6$, and $k_y = 12$ (see Supplementary Methods).

Supplementary Tables

Table A. List of sources where we collected scientific and technological words.

Source	Period	Source	Period
AccessScience	2000 – 2012	*United States Patent and Trademark Office*	1920 – 1979
Philosophical Transactions of the Royal Society	1665 – 1887	*Philosophical Transactions A, B*	1887 – 2012
Science	1880 – 2012	*Nature*	1869 – 2012
Proceedings of the National Academy of Sciences of the United States of America	1914 – 2012	*Physical Review*	1893 – 1969
Physical Review A, B, C, D	1970 – 2009	*Physical Review E*	1993 – 2009
Physical Review Letters	1958 – 2009	*Review of Modern Physics*	1929 – 2009
Physical Review Special Topics – Physics Education Research	2005 – 2009	*Physical Review Special Topics – Accelerators and Beams*	1998 – 2009

Table B. List of scientific words predicted to be type-II, which first passed the frequency of 2.0×10^{-6} in the years between 2000 and 2008 (the exact years are recorded in the second column). From Figure 1b, the chance of being type-II is estimated to be 97.1%. We assigned a category and a context of use to each word according to its common usage.

Stem	First crossing year	Corresponding word	Category	Context of use	Description
p53	2006	p53	Bio	Cancer	Tumour suppressor.
cortisol	2001	cortisol	Med	Drug	Steroid hormone. It is used as an immunosuppressive drug.
nanoparticl	2004	nanoparticle	Nano		Small particle in a nanoscale.
nanotechnolog	2006	nanotechnology	Nano		Technology to manipulate atomic- or molecular-level objects.
nanotub	2008	nanotube	Nano		Nanometer scale tube-like object, e.g., carbon nanotube.
tsunami	2006	tsunami	Geo		A series of water waves in large scale.
dataset	2001	dataset	Etc	IT	Collection of data.

Table C. Scientific words predicted to be type-II. For the frequency of 1.5×10^{-6} first passed in the years between 2000 and 2008, they are listed in a similar way to Table B.

Stem	First crossing year	Corresponding word	Category	Context of use	Description
isoform	2006	isoform	Bio	Disease	Different forms of the same protein. It is related to Mad Cow Disease.
bioethic	2007	bioethics	Bio		Ethics related to biological and medical issues.
biofilm	2000	biofilm	Bio		Group of microorganisms sticking to each other on a surface.
biomark	2006	biomarker	Med	Disease	Measurable characteristics that sense a sign of disease.
thrombo-cytopenia	2002	thrombo-cytopenia	Med	Disease	Decrease of platelets in blood.
osteoarthr	2007	osteoarthritis	Med	Aging, Disease	Degenerative joint disease.
nanotechnolog	2006	nanotechnology	Nano		Technology to manipulate atomic- or molecular-level objects.
nanoparticl	2004	nanoparticle	Nano		Small particle in a nanoscale.
nanotub	2004	nanotube	Nano		Nanometer scale tube-like object, e.g., carbon nanotube.
tsunami	2005	tsunami	Geo		A series of water waves in large scale.
holocen	2007	Holocene	Geo		Geological epoch that began at the end of the Pleistocene and continues to the present.

Table D. Scientific words predicted to be type-II. For the frequency of 1.0×10^{-6} first passed in the years between 2000 and 2008, they are listed in a similar way to Table B.

Stem	First crossing year	Corresponding word	Category	Context of use	Description
vegf	2006	VEGF	Bio	Cancer	Growth factor that stimulates blood vessel formation. Cancer related.
transferrin	2002	transferrin	Bio	Disease	Blood plasma glycoprotein that controls the level of free iron.
biofuel	2008	biofuel	Bio	Energy, Environment	Fuel produced from living organisms.
reuptak	2006	reuptake	Bio	Neurological diseases and disorders	Reabsorption of a neurotransmitter by a pre-synaptic neuron.
cd8	2007	CD8	Bio		Co-receptor for the T cell receptor, mainly expressed in cytotoxic T cells.
cyanobacteria	2000	cyanobacteria	Bio		Bacteria capable of photosynthesis.
nephropathi	2004	nephropathy	Med	Aging, Disease	Kidney disease.
biomark	2006	biomarker	Med	Disease	Measurable characteristics that sense a sign of disease.
neurologist	2007	neurologist	Med	Neurological diseases and disorders	Physician who specializes in neurology.
biomateri	2006	biomaterial	Med		Any object that interacts with biological systems.
biosensor	2007	biosensor	Med		Device for the detection of biological components.
detoxif	2001	detoxification	Med		Removal of toxic substances from a living body.
microsystem	2001	microsystem	Nano		Miniaturized device for non-electronic function.
nanoparticl	2003	nanoparticle	Nano		Small particle in a nanoscale.

Stem	First crossing year	Corresponding word	Category	Context of use	Description
nanotub	2003	nanotube	Nano		Nanometer scale tube-like object, e.g., carbon nanotube.
nanotechnolog	2005	nanotechnology	Nano		Technology to manipulate atomic- or molecular-level objects.
nanostructur	2006	nanostructure	Nano		Object of molecular or microscopic structure.

Table E. Scientific words that first passed the frequency of 5.0×10^{-7} in the years between 2000 and 2008. They are listed in a similar way to Table B. Although the list includes the words with a rather low chance of being type-II (78.5%) compared with Tables B–D (≥ 90.4%), we provide it for supplementary purposes.

Stem	First crossing year	Corresponding word	Category	Context of use	Description
h2o2	2006	H_2O_2	Bio	Aging	Simplest peroxide. Highly reactive oxygen species.
polyphenol	2006	polyphenol	Bio	Aging	Molecule with large multiples of phenol structural units. Antioxidant effect.
brca1	2007	BRCA1	Bio	Cancer	Breast cancer type 1 susceptibility protein.
isoflavon	2002	isoflavone	Bio	Cancer	A class of organic compounds, often naturally occurring, related to the isoflavonoids.
microenviron	2007	micro-environment	Bio	Cancer	Small-scale environment around cells.
vegf	2001	VEGF	Bio	Cancer	Growth factor that stimulates blood vessel formation. Cancer related.
proteasom	2004	proteasome	Bio	Cancer, Neurological diseases and disorders	Protein complex that degrades unneeded or damaged proteins. Related to cervical cancer and cystic fibrosis.
cardiomyocyt	2007	cardiomyocyte	Bio	Disease	Heart muscle. Related to cardiomyopathy.
metallo-proteinas	2005	metallo-proteinase	Bio	Disease	Protease enzyme with catalytic activity involving a metal. Used to treat periodontal disease.
ubiquitin	2002	ubiquitin	Bio	Neurological diseases and disorders	Small regulatory protein leading to protein degradation. Related to Alzheimer's and Parkinson's diseases.
cannabinoid	2002	cannabinoid	Bio	Neurological diseases and disorders	Chemical compound acting on cannabinoid receptors on cells that repress neurotransmitter release in the brain.

Stem	First crossing year	Corresponding word	Category	Context of use	Description
microglia	2006	microglia	Bio	Neurological diseases and disorders	Macrophages in the brain. Related to Alzheimer's and Parkinson's diseases.
neurogenesi	2006	neurogenesis	Bio	Neurological diseases and disorders	Process to generate neurons.
hydrogel	2000	hydrogel	Bio	Tissue engineering	Hydrophilic polymer network.
biofuel	2007	biofuel	Bio	Energy, Environment	Fuel produced from living organisms.
archaea	2007	archaea	Bio		One of the three domains of life.
aspartam	2001	aspartame	Bio		Non-saccharide, sugar substitute.
biomolecul	2006	biomolecule	Bio		Any molecule produced by a living organism.
fluorophor	2006	fluorophore	Bio		Fluorescent chemical compound.
immunohisto-chemistri	2006	Immunohisto-chemistry	Bio		Process of detecting antigens in cells. Used for diagnosis.
ligas	2006	ligase	Bio		Enzyme for the joining of two large molecules.
luciferas	2006	luciferase	Bio		Enzyme for bioluminescence.
paclitaxel	2005	paclitaxel	Med	Cancer	Mitotic inhibitor used in cancer chemotherapy.
hemo-chromatosi	2000	hemo-chromatosis	Med	Disease	Iron overload in the body.
metformin	2007	metformin	Med	Drug	Antidiabetic drug.

Stem	First crossing year	Corresponding word	Category	Context of use	Description
prodrug	2007	prodrug	Med	Drug	Drug initially administered in an inactive form, later converted to its active form in the body.
meth-amphetamin	2004	meth-amphetamine	Med	Drug, Neurological diseases and disorders	Narcotic to treat attention deficit hyperactivity disorder (ADHD).
valproat	2007	valproate	Med	Drug, Neurological diseases and disorders	Drug to treat epilepsy and bipolar disorder.
phytolith	2006	phytolith	Geo		Silica from plants. Used for archaeological and paleoenvironmental research.
plasmon	2006	plasmon	Physics		Quantum of plasma oscillation.
supersymmetri	2000	supersymmetry	Physics		Symmetry that relates two basic classes of particles, bosons and fermions.
dendrim	2006	dendrimer	Nano		Repetitively branched molecules. Potential use for drug and gene delivery.
mesopor	2004	mesopore	Nano		Material with a nanoscale pores.
nanocryst	2003	nanocrystal	Nano		Crystal structure in a nanoscale.
nanomateri	2007	nanomaterial	Nano		Material in a nanoscale.
nanoparticl	2001	nanoparticle	Nano		Small particle in a nanoscale.
nanostructur	2002	nanostructure	Nano		Object of molecular or microscopic structure.
nanotechnolog	2003	nanotechnology	Nano		Technology to manipulate atomic- or molecular-level objects.

Stem	First crossing year	Corresponding word	Category	Context of use	Description
nanotub	2002	nanotube	Nano		Nanometer scale tube-like object, e.g., carbon nanotube.
nanowir	2004	nanowire	Nano		Nanometer scale wire-like object.
tio2	2005	TiO_2	Nano		Compound used to make inorganic nanotubes.
lightwav	2002	LightWave	IT		3D graphics tool to make movies and computer games.
spywar	2005	spyware	IT		Software to steal information from computers.
radiofrequ	2000	radiofrequency	Etc.	IT	Frequency of about 3kHz to 300GHz. Noticed for recent technologies such as radio-frequency identification (RFID).
tribolog	2001	tribology	Etc.	Nano, Bio, Environmental	Study of interacting surfaces in relative motion.

Table F. *Z*-scores and *P*-values for the fraction of type-II for scientific words having each level of frequency passed between 1800 and 1919.

**Frequency level**	***Z*-score**	***P*-value**
2.0×10^{-6}	5.93	3.04×10^{-9}
1.5×10^{-6}	8.02	1.07×10^{-15}
1.0×10^{-6}	9.25	2.28×10^{-20}
5.0×10^{-7}	10.25	1.23×10^{-24}
1.0×10^{-7}	9.15	5.85×10^{-20}
1.0×10^{-8}	4.59	4.41×10^{-6}
1.0×10^{-9}	0.46	0.64

Table G. Scientific words of late bloomer candidates (type-II with rescaled FPT ≥ 2.0). Excluded are those subject to dating or OCR errors and to non-scientific use.

Stem	Corresponding word	FPT (years)	Year of birth	Rescaled FPT	Description
eudicot	eudicots	205	1802	2.96	Monophyletic clade of flowering plant.
cuprat	cuprate	188	1804	2.77	Material containing copper anions.
retinoid	retinoid	173	1809	2.73	Vitamin A.
toxicologist	toxicologist	166	1809	2.62	Professional who specializes in the poisoning of living organisms.
megafauna	megafauna	82	1926	2.61	Large or giant animal in terrestrial zoology.
microgel	microgel	75	1932	2.61	Cross-linked three-dimensional polymer networks swollen in a solvent.
phosphopeptid	phosphopeptide	54	1950	2.60	Phosphorylated peptide.
niobat	niobate	143	1846	2.56	Salt containing an anionic grouping of niobium and oxygen.
micro-architectur	micro-architecture	53	1950	2.55	Way to implement an instruction set architecture (ISA) on a computer processor.
speleothem	speleothem	52	1953	2.54	Mineral deposit formed in a cave.
endosymbiont	endosymbiont	68	1939	2.53	Organism living within the body or cells of another organism.
nematolog	nematology	91	1914	2.53	Study of nematodes.
superoxid	superoxide	170	1804	2.5	Compound including the superoxide anion.

Stem	Corresponding word	FPT (years)	Year of birth	Rescaled FPT	Description
metalloproteas	metalloprotease	48	1959	2.48	Protease enzyme involving a metal in its catalytic activity.
trans-glutaminas	trans-glutaminase	48	1958	2.48	Enzyme that links an amine group and glutamine.
steatosi	steatosis	165	1835	2.47	Abnormal retention of lipids within a cell.
autophagi	autophagy	165	1825	2.46	Intracellular degradation of unnecessary or dysfunctional cellular components.
agaros	agarose	149	1819	2.45	Polysaccharide material extracted from seaweed.
allelopathi	allelopathy	68	1931	2.45	Biochemical interactions between organisms to affect their growth, survival, and reproduction.
vasculopathi	vasculopathy	53	1949	2.44	Disorder of blood vessels.
gapdh	GAPDH	47	1959	2.43	Enzyme involved in glycolysis.
spallat	spallation	147	1810	2.43	Ejection of fragments from a material by impact or stress.
lipodystrophi	lipodystrophy	94	1910	2.42	Abnormal or degenerative condition of adipose tissues.
cryptolog	cryptology	163	1805	2.41	Technique for secure communication in the presence of third parties.
glucokinas	glucokinase	57	1948	2.39	Enzyme to phosphorylate glucose.
discoideum	*Dictyostelium discoideum*	163	1812	2.35	Soil-living amoeba.
xenotransplant	xenotransplant	61	1937	2.35	Animal tissue or organ transplant in a human patient.

Stem	Corresponding word	FPT (years)	Year of birth	Rescaled FPT	Description
isoflavon	isoflavone	70	1928	2.34	A class of organic compounds, often naturally occurring, related to the isoflavonoids.
azoospermia	azoospermia	128	1870	2.33	Medical condition of a man without any measurable level of sperms.
selenoprotein	selenoprotein	105	1902	2.32	Protein including a selenocysteine.
thermotherapi	thermotherapy	135	1873	2.32	Application of heat to the body for health and medical purpose.
primatologist	primatologist	74	1927	2.31	Professional who studies primates.
biofuel	biofuel	58	1940	2.28	Fuel produced from living organisms.
ecosystem	ecosystem	136	1817	2.28	Community of living organisms in the environment.
bioenergi	bioenergy	68	1928	2.27	Renewable energy from biological sources.
dyslipidemia	dyslipidemia	55	1946	2.27	Abnormal amount of lipids in the blood.
microsensor	microsensor	54	1948	2.26	Sensing device of small size.
pervapor	pervaporation	74	1918	2.26	Separation of liquid mixtures through a non-porous or porous membrane.
audiometri	audiometry	141	1809	2.23	Branch of audiology for measurements of hearing acuity.
polyomavirus	*Polyomavirus*	43	1959	2.22	Oncogenic virus.
recombinas	recombinase	43	1959	2.22	Enzyme for genetic recombination.

**Stem**	**Corresponding word**	**FPT (years)**	**Year of birth**	**Rescaled FPT**	**Description**
transesterif	transe-sterification	59	1938	2.21	Exchange of organic groups in an ester and an alcohol.
asteracea	Asteraceae	147	1835	2.2	Aster, daisy or sunflower family.
desulfovibrio	*Desulfovibrio*	57	1937	2.20	Sulfate-reducing bacteria.
microcav	microcave	52	1944	2.2	Structure to confine light to small volumes by resonant recirculation.
midgut	midgut	131	1810	2.17	Part of the embryo to develop into the intestines.
paleo-anthropolog	paleo-anthropology	91	1907	2.17	Study of ancient humans as found in fossils.
theropod	theropod	103	1886	2.14	Suborder of dinosaurs.
homoplasi	homoplasy	126	1871	2.12	Independent evolution of similar features in different taxa.
aerogel	aerogel	69	1918	2.11	Gel of which liquid component has been replaced by gas.
retinol	retinol	128	1842	2.11	Vitamin A.
polyhedra	polyhedra	145	1802	2.09	Three dimensional geometrical object with flat faces and straight edges.
bloodstream	bloodstream	124	1817	2.08	Blood flow through the circulatory system.
microcentrifug	microcentrifuge	70	1922	2.08	Equipment that spins liquid samples at high speed.
phytas	phytase	90	1909	2.05	Phosphatase enzyme that hydrolyses phytic acid.

Stem	Corresponding word	FPT (years)	Year of birth	Rescaled FPT	Description
capsaicin	capsaicin	106	1877	2.02	Component of chili peppers.
angiopathi	angiopathy	124	1852	2.00	Disease of the blood vessels.

Supplementary Methods

1. Dataset

1.1. *Google Books Ngram Corpus*

We obtained the annual data of *n*-gram counts contained in the English section of the *Google Books Ngram Corpus* Version 2 which spans 8,116,746 books published over the last five centuries [1]. A 1-gram is a string of characters uninterrupted by a space, e.g., a word or number. An *n*-gram is a sequence of 1-grams, e.g., *n*=3 for a phrase with three words. We here focus on 1-grams for simplicity of analysis. A sample of the data is given below (from the data file "googlebooks-eng-all-1gram-20120701-w.csv"):

work	2000	7285922	100673
work_VERB	2000	1848009	93981
work_NOUN	2000	5377995	99002

The first row shows that in 2000, the word "work" occurred 7,285,922 times in 100,673 different books. The second and third rows show that in the same year, the word "work" occurred 1,848,009 times as a verb, and 5,377,995 times as a noun. Relative frequency of a 1-gram is defined as the number of occurrences of the 1-gram in a given year divided by the total number of 1-grams in that year.

1.2. Preprocessing

In order to treat different inflectional forms (e.g., singular and plural) of the same word stem as equivalent in their essential meaning, we integrated such forms systematically by *Porter Stemming Algorithm* [2] when computing the 1-gram frequency. We also limited our analysis to data in the years between 1800 and 2008 because the amount of data before 1800 is not sufficient to obtain statistically meaningful results [3]. In addition, every 1-gram frequency in the years 1899 and 1905 was replaced by the average for that 1-gram from ±1 years, as the *Google Books Ngram Corpus* occasionally assigned 1899 or 1905 to books of unknown publication dates [3]. For any 1-gram that appeared in year t but not in $t-1$ and $t+1$ to $t+10$ against what expected from the usual patterns (Figure A), we set its frequency in t to zero to avoid possible errors from the optical character recognition (OCR) processes.

1.3. Identification of scientific and technological words

How do we know whether a given 1-gram belongs to the vocabulary of science and technology? One simple way is to check whether it matches any word in a published science dictionary. We created a list of scientific and technological words (1-grams) from an online science dictionary "*AccessScience*" [4]. Because the list itself may be biased toward words in common use today, we added words from other various sources including those used in the past as well (Table A): we extracted words from patent grant texts in the *United States Patent and Trademark Office* data provided by Google [5] and from article titles in a number of scientific journals. Among those words, only nouns were selected. A word was considered to be a noun, if it was used as a noun in

more than 90% of its total usage in the year 2000 (e.g., 5,377,995 / 7,285,922 = 73.8% usage as a noun for "work" in section '*Google Books Ngram Corpus*'). We filtered out words with the year of birth < 1800 to make them consistent with section 'Preprocessing'. Then, we arranged the remaining words in descending order of usage within their respective sources. For most cases, the words of high usage within the sources were likely to be scientific and technological words. By manual inspection of randomly-sampled words (≥ 10% coverage for journals, ≥ 1% coverage for patents) along the descending order of usage level within each source, we selected all words of the usage level having at least an 80% chance of being scientific and technological words which are not used in too broad a context. If this cutoff covered all words occurring in that source, then we excluded words used only once in the source. In total, we obtained 7,855 scientific and technological words from the dictionary, patents, and journals.

1.4. Connection between word usage and events in society

One may ask about how word usage is related to the empirical events in society. We here present several examples in response to such questions. The original study of *Google Books Ngram Corpus* [3] reported that the boost of a word in its frequency can reflect the increasing impact of the relevant event on society. For example, peaks in "influenza" correspond to the dates of known pandemics [3]. Additionally, various studies in sociolinguistics have paid attention to connections between, e.g., social structures and word usage [6]–[7], urbanized population and word usage [8], and events in society and coherent changes in word usage [9]. Those studies seem to support our assumption that the frequency of a scientific word is indicative of the actual impact of the scientific concept on society."

2. Data analysis

2.1. Determination of f_c, FPT, lifetime, and peak

The cutoff frequency f_c defines the threshold above which a 1-gram can be roughly considered to be common in society. A proper choice of f_c is important as the quantification of first passage time and lifetime (see below) depends on it. We chose $f_c = 10^{-7}$ since 1-grams with frequency $> 10^{-7}$ are easily found in published dictionaries [3]. In 2000, there were 79,691 word stems (corresponding to ~200,000 1-grams) with frequency $> 10^{-7}$ (Figure B). Our main results presented in this work, however, do not qualitatively change as long as $10^{-8} \leq f_c \leq 2\times10^{-7}$. For a given 1-gram, first passage time (FPT) is defined as years to cross f_c in frequency since the birth of the 1-gram, lifetime is defined as years between the first and the last year the frequency was above f_c, and peak is defined as the highest frequency of the 1-gram over time. Specifically, we define lifetimes to 1-grams, which are under the frequency f_c for at least 10 years until the year 2008, since they are rarely expected to bounce back (Figure A). Figure C illustrates the definitions of FPT, lifetime, and peak.

2.2. Characterization of different 1-gram types

Most 1-grams could be classified into the following three types. Type-I has 1-grams with well-defined finite lifetimes (section 'Determination of f_c, FPT, lifetime, and peak'). Type-II shows a

lifetime to a distinctively long extent beyond the time frame, so the exact lifetime cannot presently be defined. Type-III, unlike types-I and -II, never had a frequency higher than f_c. One may claim that the distinction between type-I and type-II was merely based on the limited period of observation allowed in our current dataset. Although the distinction was made in a rather heuristic way, we did observe a more fundamental difference between type-I and type-II. Figure D shows the probability density function (PDF) of the frequency for each type of 1-grams in a given year. While the PDFs for type-I and type-II initially overlap, the difference between them grows over time as the PDF of type-II shifts to higher frequency ranges. The growing difference can be quantified by tracking the average and median frequencies of each type over the years, as shown in Figure E. While the average and median frequencies of type-I stay almost steady, the same statistics of type-II keep increasing. The results indicate an intrinsic difference between types-I and -II, manifested in their frequency growth patterns.

2.3. Predictability

Type-II includes scientific words prevailing in society longer than the other types. Thus, by identifying type-II scientific words at a relatively early stage, we can predict which words will be promising in the future. As demonstrated in Figure E, the frequency of a type-I word tends to stay at a low level, while that of a type-II word continuously grows. This fact implies that if we identify the scientific words whose frequency exceeds a sufficiently high level, many of them will be type-II. Figure F indeed shows that the higher the level of frequency exceeded, the more likely the word belongs to type-II. It also shows that the probability of being type-II varies slightly across the years when the words passed a particular level of frequency. This raises the question of which years are appropriate to choose to estimate the precision of type-II identification. The period of the years should be long enough for a reliable statistical analysis and the years should be old enough for a clear distinction between type-I and type-II in 2008. We selected the period of years between 1800 and 1919, which leaves 89 years until the end year of our dataset, and this 89-year period is longer or comparable to the typical lifespan of a human being.

For the period 1800–1919, the relationship between the level of frequency exceeded and the probability of being type-II in 2008 is presented in Figure 1b. Accordingly, we made a list of scientific words predicted to be future type-II based on the level of frequency passed in the years between 2000 and 2008 (Tables B–E). All entries were classified into respective categories, and we filtered out the words used in too broad a context, not necessarily in a scientific context.

2.3.1. Significance test

To test the statistical significance of the relation between the level of frequency passed and the probability of being type-II, we performed a two-sided Z-test under the null hypothesis that there is no association between the frequency level and the probability of type-II, resulting in their correlation merely by chance. For this analysis, we calculated expected values and standard deviations from the null distributions. Among N scientific words (1-grams) in total, let q be the fraction of words over a certain frequency level and r be the fraction of type-II. The expected number of type-II over the frequency level is Nqr and the variance is $Nqr(1-r)$. The central limit theorem ensured that this null distribution converged well to the Gaussian distribution, giving a Z-score as well as a P-value (Table F).

2.3.2. Internet webpage volume

To test the validity of our type-II prediction results against an up-to-date independent dataset, we used the Google web search engine that showed the Internet webpage volumes updated annually between 2008 and 2013 for the words of our search queries (accessed in February and March 2014). Because Google provides search results using a stemming algorithm, we submitted the singular forms of the words instead of the word stems themselves. Because Google does not permit automatic search queries by web robots, we manually submitted (i) the type-II-predicted scientific words in Tables B–D, and (ii) their counterparts, randomly-selected from the scientific words that first reached any frequency $\leq 2\times10^{-6}$ between 2000 and 2008. For the normalization in Figure 1c, we used 100 random words from (ii). For the control group against (i) in Figure 1d, we used 100 random words from (ii) not overlapping with (i). In Figure 1d, the comparison between the search queries for (i) and for the control group shows that the prediction results also work for the webpage volumes since 2008, although the prediction itself is based on the 1-gram data between 2000 and 2008.

2.4. Rescaling of FPT and lifetime

Figure G gives a detailed visualization of the results in Figure 2a: for each set of type-I 1-grams born in the same year, the complementary cumulative distribution functions (CCDFs) of FPT, lifetime, and their rescaled values (FPT and lifetime divided by their respective averages from the same year of birth) are plotted. Overall FPT and lifetime were getting shorter over the past years, as the CCDFs of FPT and lifetime were getting steeper as the years passed. Rescaled values lead to a collapsing of their CCDFs from different years into an approximately single curve, indicating nearly equivalent patterns are followed across years for FPT and lifetime. The rescaling doesn't only work for all type-I 1-grams, but also separately for the type-I scientific words among them (Figure H).

2.5. Relations between FPT, lifetime, and peak

This section discusses the unique features of scientific words manifested in the relations between FPT, lifetime, and peak. For FPT and lifetime in this section, we use their rescaled values (section 'Rescaling of FPT and lifetime') unless specified.

2.5.1. Adjusted density plot

To find the correlation between two quantities, *x* and *y* in the linear scale, we first take a small window of size $b_x \times b_y$, place the lower left corner of the window at the starting (smallest) points of *x* and *y* (x_{min}, y_{min}), measure the density of data points inside the window, and assign the value to the lower left corner. We repeat the same procedure after shifting the position of the window by b_x/k_x along the *x*-axis or b_y/k_y along the *y*-axis until the entire *xy*-plane is spanned (k_x and k_y are constants). If one axis (say *x*) is in the logarithmic scale, the density at each position is calculated in a similar way except that the window is shifted in the *x*-direction by multiplying $(i_x)^{1/k_x}$ to the *x*-coordinate and the window length along the *x*-axis increases by the same factor. Finally, we normalize every density at each *x* relative to the maximum across the *y*-axis. We call this density "adjusted density", which is suited for clarifying the dependence of *y* on *x* when plotted on the *xy* plane.

2.5.2. FPT and lifetime

Figure I (same as Figure 2b and c) shows the density plot between FPT and lifetime, for scientific words (left) and an entire set of 1-grams (right) in type-I. For scientific words, FPT and lifetime are negatively correlated, with a transition at FPT~1.2 giving rise to a sudden appearance of lifetime~2.0 (Pearson's Chi-squared test, P = 4.3×10^{-47}). For an entire set of 1-grams, there is no such transition.

2.5.3. Peak and lifetime

Figure J shows the density plot between peak and lifetime for scientific words (left) and an entire set of 1-grams (right) in type-I. At small values of peak for scientific words, lifetimes are mostly short. As peak increases, a sudden leap from short to long lifetime is observed at peak ~ 5×10^{-7}. This transition barely occurs for an entire set of 1-grams, at much larger peak (11.3 times larger) than for scientific words.

2.5.4. FPT and peak

Figure K shows the density plot between FPT and peak for scientific words (left) and an entire set of 1-grams (right) in type-I. FPT and peak have negative correlation.

2.5.5. Significance test

To test the statistical significance of sudden leap into ~2.0 in lifetime at FPT~1.2 for type-I scientific words, we constructed a 2× 2 contingency table displaying the numbers of the words at FPT ≥ 1.2 and < 1.2, and lifetime ≥ 2.0 and < 2.0. Then, we computed the Pearson's Chi-square value and a P-value based on a Chi-square distribution with 1 degree of freedom, with a null hypothesis that there is no association between FPT and lifetime.

3. Model description

To build a mechanistic model to account for our observation, we considered the three key factors in the spread of science and technology – preferential adoption, homophily, and fitness, as described in the main text. In this section, we explain further details of how the model accommodates these factors. The model consists of N agents where individual agents represent various forms of social units to invent and adopt items. The items are transmitted from agent to agent. We assume that the adopted ranges of such items are projected into the actual usage levels of the corresponding words in the 1-gram dataset [3].

3.1. Homophily

Each agent is assigned ε, which characterizes the level of involvement in specialized areas. In general, ε can be a vector with real-number components. For the simplicity of our model, here ε is a scalar binary number: $\varepsilon = 1$ if the agent belongs to the scientific community, otherwise, $\varepsilon = 0$. In other words, agents such as scientists, engineers, scientific journalists, research institutes, and scientific publishers can take $\varepsilon = 1$, and we call them simply 'scientists' in our model. Scientists occupy only a small fraction of the whole system, with a certain chance of being a scientist (equal

to ρ) given to each agent at the beginning of the simulation. Once ε has been determined to be either $\varepsilon = 1$ or 0 for each agent, it never changes during the simulation. To consider the effect of homophily, we introduce a weight function for every pair of agents, $w(|\varepsilon_i - \varepsilon_j|)$, which captures how influential agents *i* and *j* in the pair are to each other in the spread of innovation. $w(|\varepsilon_i - \varepsilon_j|)$ should be a decreasing function of $|\varepsilon_i - \varepsilon_j|$ and we chose the form $w(|\varepsilon_i - \varepsilon_j|) = \exp[-(\varepsilon_i - \varepsilon_j)^2]$.

3.2. Preferential adoption and homophily

When agent *i* adopts another *j*'s item *q*, preferential adoption and homophily work as the following function, $p(q, i)\times p(q, j)$, where

$$p(q,m) = \sqrt{\frac{\sum_r \delta(q,r) w(|\varepsilon_m - \varepsilon_r|)}{\sum_r w(|\varepsilon_m - \varepsilon_r|)}}\ .$$

Here, $\sum_r$ denotes the sum over all agents in the system and $\delta(q, r)=1$ if agent *r* holds item *q*, otherwise, $\delta(q, r)=0$. $w(|\varepsilon_m - \varepsilon_r|)$ comes from section 'Homophily'. A square root appears in $p(q, m)$ because it makes $p(q, i)\times p(q, j)$ linearly proportional to the population having item *q* in the case that ε's are identical for all agents.

3.3. Network for information spread

Adoption of new items takes place through direct information spread between agents. For the simulation results presented in this study, the global network topology of such information channels connecting different agents was set following the Erdős–Rényi model [10]. Specifically, we used a $G(N,p_{ER})$ model where each agent is randomly linked to another with probability p_{ER} [11]. To avoid generating isolated agents, we took $p_{ER} > \ln(N)/N$.

We also considered another network model, the static model of scale-free networks [12], which is known to produce a fat-tailed, power-law degree distribution in contrast to the Erdős–Rényi model. For the degree exponent between 2.0 and 3.0 (other parameters set equal to those of Figure 3a–d), we found that our main results did not much change with the selection of this network topology.

3.4. Fitness

To each invented item, we assign fitness λ, which gives the intrinsic differences between items in their adoption rates.

3.4.1. Gaussian distribution

Provided that fitness λ is a sum of numerous uncorrelated properties of an item, one can assume that the fitness distribution follows the Gaussian distribution $\Lambda_g(0.5, \sigma) \sim \exp[(\lambda-0.5)^2/2\sigma^2]$, whose domain is centred at 0.5 and bounded by 0 and 1. In this case, we consider the following function contributing to the probability that a new item q_j with fitness λ_{q_j} replaces an old item q_i with fitness λ_{q_i} in its adoption:

$$f(\lambda_{q_j}-\lambda_{q_i})=\frac{1}{2}+\left(\frac{\lambda_{q_j}-\lambda_{q_i}}{2}\right) \quad .$$

3.4.2. Power-law distribution

Alternatively, one can assume that the fitness distribution follows a fat-tailed distribution such as a power-law, Λ_P (γ, x_{min}) ~ $(x/x_{min})^{-\gamma}$, whose domain is bounded by 1 and 11. In this case, we consider the following function contributing to the probability that a new item q_j with fitness λ_{q_j} replaces an old item q_i with fitness λ_{q_i} in its adoption:

$$f(\lambda_{q_j}-\lambda_{q_i})=\frac{1}{2}+\left(\frac{\lambda_{q_j}-\lambda_{q_i}}{10}\right)^{\beta} \quad if \quad \lambda_{q_j}\geq\lambda_{q_i} \ ,$$

$$f(\lambda_{q_j}-\lambda_{q_i})=\frac{1}{2}-\left(\frac{\lambda_{q_i}-\lambda_{q_j}}{10}\right)^{\beta} \quad if \quad \lambda_{q_j}<\lambda_{q_i} \ .$$

3.5. Update rule

In our model, every agent has L distinct items at every instant. At every time step, a new item is introduced by randomly-selected agent i with probability α, and is assigned the category simply by following agent i's specialty ε_i (section 'Homophily'). The new item randomly replaces one of the agent i's old items in the same category as the new one. If there is no such item in the same category, any old item of agent i is randomly chosen and replaced. The new item has fitness with the probability distribution mentioned in section 'Fitness'.

Next, we randomly select a pair of agents j and k in direct contact through pre-assigned information channels (section 'Network for information spread') and their items q_j and q_k belonging to the same category. If agents j and k have no items in the same category, any pair of their items is selected. Then, agent j adopts item q_k by replacing item q_j with the following probability, provided that agent j has never adopted item q_k before:

$$P(q_j,q_k,j,k)=f(\lambda_{q_k}-\lambda_{q_j})\times p(q_k,j)\times p(q_k,k),$$

where λ_q is item q's fitness, and each function is described in the previous sections. If $P(q_j,q_k,j,k)$ is smaller than 0 (larger than 1), we consider it to be 0 (to be 1). At every $N\times L$ repetitions of the above steps, the frequencies of all items in the system are recorded. The frequency of an item is defined as the ratio of the item's copy number to the total counts of items (= $N\times L$) in the system. Here, we use such $N\times L$ repetitions of the steps as the arbitrary unit of time to measure the FPT and lifetime of items.

3.6. Initialization

After the system is set up with given parameters, we start with N agents having no items. We run the simulation as described in section 'Update rule', except that a transmitted (newly generated) item is appended to the receiving (producing) agent's item list if the list contains fewer than L distinct items. If the receiving (producing) agent already has L distinct items, then one of them is replaced with the transmitted (newly generated) item according to the rules in section 'Update

rule'. The initialization process is complete once every agent has L distinct items, and the simulation time starts at that moment.

3.7. Ergodicity

In section 'Data analysis', all statistics for the 1-gram data were obtained from the long time series data. For the model analysis here, we use the ensemble results assembled from multiple simulations rather than use the results from a single long simulation, to save simulation times. Simulations for each ensemble were performed under the same model parameters but can have different initial conditions and network connectivity due to the randomness in the initialization process. One may question the validity of using such ensemble results instead of results from a sheer long-time simulation. We claim that our model is ergodic enough so that both ensemble and long-time results give almost equivalent patterns. Two Erdős–Rényi networks with equal p_{ER} do not have much statistical difference in their structural properties when the network size is large enough [11], so their dynamical properties would not be much different either. Moreover, most items cannot survive over the frequency f_c in the system for longer than 50000 steps, and within 100000 steps all items of the system fall below f_c and are effectively replaced by the new, not leaving much trace of the past. Therefore, a long simulation of our model would be nearly equivalent to an ensemble of different simulations.

4. Model results

The simulation of our model shows, whether for the scientific category or not, the existence of type-II-like items having distinctively longer lifetimes than the others (Figures L–N; see also section 'Distinct dynamics of type-I and type-II in their adoption'). They appear even if all agents and items are assigned the same ε and the same λ, respectively, indicating that preferential adoption is sufficient for the existence of type-II (Figure N for the same λ case). However, homophily and fitness effects are also important to explain the observed patterns in scientific words, as discussed below.

4.1. Relation between FPT and lifetime

In Figures L-N, we show density plots between FPT and lifetime for different forms of fitness distributions, which supplement the results in Figure 3a and b. We checked the cases of the power-law (Figure L), Gaussian (Figure M), and Dirac delta (i.e., identical fitness for all items; Figure N) fitness distributions. In the figures, each top panel is a zoomed-in view of the bottom panel, a region below the boundary made by the constant level of the sum of FPT and lifetime. This boundary imitates the limits of the total time frame of the real 1-gram data, defining a type-I case that indeed captures the FPT–lifetime relationship of type-I observed empirically in Figure 2b and c.

For all three different fitness distributions, we could identify the range of parameters in which (i) type-I and type-II items are clearly distinguishable and (ii) type-I scientific items exhibit the sudden transition of lifetime across FPT. If we don't consider preferential adoption, homophily, and fitness for our model, then the functional form of $P(q_j, q_k, j, k)$ in section 'Update rule' is changed into $P(q_i, q_j, i, j) = \theta$, where θ is an arbitrary constant. In this case, the feature (i) is observed at a very narrow range of θ, e.g., only at $\theta \sim 0.01$ in the same condition as Fig. 3a–d. If

we now consider preferential adoption, the feature (i) appears easily without such parameter fine-tuning. However, preferential adoption alone is not enough for the feature (ii), as the feature (ii) does not appear if all agents have identical ε's. Therefore, for the features (i) and (ii), preferential adoption and homophily are both important. It is noteworthy that features (i) and (ii) can be produced, even with the Dirac delta distribution of the fitness. Nonetheless, fitness is also important in our model, as the negative correlation between FPT and lifetime in the regime of (rescaled) long lifetime ≥2.0 in Figure 2b after the transition, herein called feature (iii), cannot be reproduced under the Dirac delta distribution of fitness (Figure N). Therefore, the three fundamental components in the model – preferential adoption, homophily, and fitness – are important to explain the observed patterns in scientific evolution.

For Figure 3a–d, we used the power-law fitness distribution with parameters $\gamma = 2.0$, $\beta = 1/4$, $N = 4{,}096$, $L = 10$, $p_{\mathrm{ER}} = 0.1024$, $\rho = 0.2$, $\alpha = 0.0001$, $f_{\mathrm{c}} = 0.00025$. We found that such model outcomes do not qualitatively change, at least in the following parameter ranges: (given $N = 4{,}096$, $L = 10$, and $\beta = 1/4$) $0.1 \le \rho \le 0.3$, $0.00001 \le \alpha \le 0.001$, $0.0002 < f_{\mathrm{c}} < 0.0003$, $0.0256 \le p_{\mathrm{ER}} < 0.2048$ [for (ii); power-law fitness distribution] or $0.0064 \le p_{\mathrm{ER}} < 0.01$ [for (ii); Gaussian and Dirac delta fitness distributions], $2 \le \gamma \le 2.4$ [for (iii); power-law fitness distribution] or $0.07 \le \sigma \le 0.13$ [for (iii); Gaussian fitness distribution].

4.1.1. Significance test

To test the statistical significance of the sudden transition of lifetime across FPT for type-I scientific items, we conducted an analysis similar to section 'Significance test' in 'Data analysis': in Figure 3a, an abruptly long lifetime ~ 2,000 appears at FPT ~ 5,000. We constructed a 2×2 contingency table displaying the numbers of the words at FPT ≥ 5,000 and < 5,000, and lifetime ≥ 2,000 and < 2,000. Then, we computed the Pearson's Chi-square value and a P-value based on a Chi-square distribution with 1 degree of freedom, with a null hypothesis that there is no association between FPT and lifetime ($P = 5.4\times10^{-22}$).

4.2. Distinct dynamics of type-I and type-II in their adoption

The right panels of Figures L–N show clear gaps between short and long lifetimes of non-scientific items, giving a straightforward way to split type-I and type-II at lifetimes ~ 12,000, 8,400, and 8,000 for Figures L-N, respectively. We assume that these values of lifetime to split type-I and type-II are approximately equal for both non-scientific and scientific items. Based on this assumption, we split type-I from scientific items along the boundaries defined in the legends of Figures L-N (see also section 'Relation between FPT and lifetime'). One may question the validity of this classification scheme of type-I and type-II for scientific items, as the left bottom panels of Figures L-N show less clear gaps between type-I and type-II than the right bottom panels. Nonetheless, we were able to demonstrate that type-I and type-II scientific items are qualitatively different in their dynamics.

Figure O shows the probability distributions of $\Delta t_f = t_f' - t_f$ for type-I and type-II scientific items, where t_f' (t_f) of each item denotes the last time that item's frequency outside (inside) the scientific community fell below f_{c}. $\Delta t_f > 0$ (< 0) indicates that the item has been in longer common use outside (inside) than inside (outside) the scientific community. In Figure O, we observe that type-I and type-II tend to occupy different regimes of Δt_f: $\Delta t_f < 0$ for type-I and $\Delta t_f > 0$ for type-II. In other words, type-II scientific items tend to survive in the outer society even though they are no

longer active within the scientific community. The adoption of type-I scientific items shows the opposite trend, largely driven by the internal dynamics of the scientific community itself. In conclusion, the simulation results demonstrate the fundamental difference between type-I and type-II in their dynamics during adoption, supporting the validity of our classification scheme for type-I and type-II. For this analysis, we excluded the items whose frequency either outside or inside the scientific community never exceeded f_c, because of their ill-defined t_f' and t_f. These items would not be well found near the boundaries between type-I and type-II, so excluding them would not distract the rigorous examination of the difference between these two types.

4.3. Effect of fitness on lifetime and peak

In our model, the spread of an item depends on its fitness as well as social effects. The latter effects do not always favor the spread of a higher-fitness item because they may amplify random fluctuations in the item's spread and strengthen the spread of the item in the majority regardless of its fitness. In this section, we present simulation results showing how critical fitness is in determining the long-term fate of individual items – lifetime and peak.

Figure P shows the averages of lifetime and peak steadily increasing over fitness, but also the large variability out of this average trend. In Figure 3c and Figure Q, we use the coefficient of variation (CV) as an indicator of the variability. CV is defined as the ratio of standard deviation to mean. Figure Q shows that the variability of lifetime and peak increases non-monotonically across fitness, reaching the maximum at the intermediate level of fitness. In other words, the long-term fate of scientific items is less variable at low and high fitness, and actually type-II and type-III have distributions biased to these fitness regimes (type-II for high fitness and type-III for low fitness; Figures Q–R).

5. Late bloomers: effect of fitness on FPT

Common intuition suggests that FPT and fitness should be anti-correlated. On the contrary, the simulation results clearly show the positive correlation between them for types-I and -II scientific items (Figure S). These counter-intuitive results can be explained by the fact that high fitness helps the science survive long periods of frequency < f_c, allowing for long FPT as well as short FPT (Figure T). In contrast, low-fitness science is difficult to survive unless it initially spreads fast, either having short FPT or falling to type-III (Figure T). The existence of high fitness, long FPT science reminds us of the concept 'late bloomers'.

We found that such model outcomes do not qualitatively change, at least in the following parameter ranges: (given $N = 4{,}096$, $L = 10$, and $\beta = 1/4$) $0.1 \leq \rho \leq 0.3$, $0.00001 \leq \alpha \leq 0.001$, $0.0002 < f_c < 0.0003$, $0.0064 \leq p_{ER} < 0.8192$ (power-law fitness distribution) or $0.0064 \leq p_{ER} < 0.01$ (Gaussian fitness distribution), $2 \leq \gamma \leq 2.4$ (power-law fitness distribution) or $0.07 \leq \sigma \leq 0.13$ (Gaussian fitness distribution).

The above findings raise the possibility that scientific words with very long but finite FPT in the *Google Books Ngram Corpus* dataset can be good candidates for late bloomers with high fitness. We listed in Table G such late bloomer candidates from type-II scientific words with rescaled FPT ≥ 2.0. For this, we manually excluded the words involving dating or OCR errors, and non-scientific use.

5.1. Significance test

To test the statistical significance of a positive correlation between FPT and fitness, we performed a two-sided *Z*-test under the null hypothesis that there is no association between FPT and the fraction of scientific items with high fitness >10.5 (Figure 3d; power-law fitness distribution with $\gamma = 2.0$, $\beta = 1/4$, $N = 4{,}096$, $L = 10$, $p_{ER} = 0.1024$, $\rho = 0.2$, $\alpha = 0.0001$, $f_c = 0.00025$). We calculated an expected value and a standard deviation from the null distribution. For types-I and -II scientific items, let q be the fraction of FPT > 10000 and r be the fraction of fitness > 10.5. If there are N items in total, the expected number of items with fitness > 10.5 among those with FPT > 10000 is Nqr and the variance is $Nqr(1-r)$. The central limit theorem ensured that this null distribution converged well to the Gaussian distribution, giving a *Z*-score as well as a *P*-value.

6. Evolution of other fields

Our model predicts that other innovative fields such as food and art have similar features to science in FPT-lifetime relation, as demonstrated in Figure 4. However, one of the sources from which we collected food-related words [13] contained 43 (out of 236) type-I words overlapping with those analysed for scientific evolution. To avoid any possible artifact in Figure 4a made by such overlapping, we repeated the analysis after excluding the overlapping words and again found a result similar to Figure 4a (Figure U; Pearson's Chi-squared test, $P = 4.9\times10^{-7}$).

6.1. Significance test

To test the statistical significance of the sudden leap in lifetime across FPT in Figure 4 and Figure U, we conducted an analysis similar to section 'Significance test' in 'Relations between FPT, lifetime, and peak'.

Supplementary References

1. Lin Y, Michel J-B, Aiden EL, Orwant J, Brockman W, et al. (2012) Syntactic annotations for the Google Books Ngram Corpus. Proceedings of the 50th Annual Meeting of the Association for Computational Linguistics: 169–174.
2. Porter MF (1980) An algorithm for suffix stripping. Program 14: 130–137.
3. Michel J-B, Shen YK, Aiden AP, Veres A, Gray MK, et al. (2011) Quantitative analysis of culture using millions of digitized books. Science 331: 176–182.
4. AccessScience. Available: http://www.accessscience.com/. Accessed: 24 February 2011.
5. Google United States Patent and Trademark Office Bulk Downloads. Available: http://www.google.com/googlebooks/uspto.html. Accessed: 16 October 2012.
6. Labov W (1966) The linguistic variable as a structural unit. Wash Linguist Rev 3: 4–22.
7. Chambers JK (1997) Sociolinguistic theory. Oxford: Blackwell.
8. Greenfield PM (2013) The changing psychology of culture from 1800 through 2000. Psychol Sci 24: 1722–1731.
9. Wijaya DT, Yeniterzi R (2011) Understanding Semantic Change of Words Over Centuries. DETecting and Exploiting Cultural diversiTy on the social web: 35–40."
10. Erdős P, Rényi A (1960) On the evolution of random graphs. Publ Math Inst Hungar Acad Sci 5: 17–61.
11. Gilbert EN (1959) Random graphs. Ann Math Stat 30: 1141–1144.
12. Goh K-I, Kahng B, Kim D (2001) Universal behavior of load distribution in scale-free networks. Phys Rev Lett 87: 278701.
13. Bender DA (2009) A dictionary of food and nutrition. 3rd ed. Oxford: Oxford University Press.
